# Mind the gap: obtaining reliable sleep estimates and the diagnostic value of sleep discrepancy in individuals with Alzheimer's disease and Lewy body disease

**DOI:** 10.1002/alz.71377

**Published:** 2026-04-23

**Authors:** Victoria Grace Gabb, Jonathan Blackman, Hamish Duncan Morrison, Nicholas Turner, Amanda Heslegrave, Elizabeth Coulthard

**Affiliations:** ^1^ Bristol Medical School University of Bristol Bristol UK; ^2^ Bristol Brain Centre Southmead Hospital North Bristol NHS Trust Bristol UK; ^3^ NIHR Bristol Biomedical Research Centre Bristol UK; ^4^ Institute of Neurology University College London London UK; ^5^ UK Dementia Research Institute University College London London UK

**Keywords:** actigraphy, Alzheimer's disease, dementia, EEG, electroencephalography, Lewy body disease, objective, older adults, sleep, sleep discrepancy, subjective

## Abstract

**INTRODUCTION:**

Objective sleep disturbances, including short and fragmented sleep, are observed in neurodegenerative diseases. However, subjective sleep disturbances are inconsistently reported. Improved understanding of objective and subjective sleep estimation is needed to tailor sleep interventions.

**METHODS:**

Baseline subjective habitual sleep was compared to objective sleep measured by actigraphy averaged over 8 weeks in 20 patients with mild cognitive impairment (MCI) or dementia due to Alzheimer's disease (AD) or Lewy body disease (LBD) and 20 healthy older adults. Discrepancies between objective and subjective sleep parameters were used to predict cohort membership (AD, LBD, or control).

**RESULTS:**

Participants with AD and LBD estimated lower sleep disturbance than actigraphy. Subjective sleep quality was poorest in LBD and highest in AD. Subjective sleep and subjective‐objective sleep discrepancy discriminated between cohorts with 80% accuracy.

**DISCUSSION:**

Subjective and objective sleep differ and both should be measured in MCI and dementia. Sleep discrepancy may have diagnostic utility.

## Highlights

- Patients with probable AD reported good sleep quality.
- Patients with probable LBD reported poor sleep quality.
- Both patient groups demonstrated positive sleep discrepancy.
- Subjective sleep quality and sleep discrepancy discriminated between groups.
- Sleep disturbance may be underestimated by subjective sleep measures.

## Graphical abstract

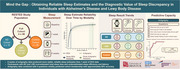

## 1 BACKGROUND

Sleep disturbances are increasingly considered risk factors for and symptoms of cognitive decline and dementia.^1^, ^2^, ^3^, ^4^ Polysomnography, the gold standard for sleep assessment, demonstrates shorter sleep duration, more fragmented sleep, and reduced rapid eye movement (REM) sleep in individuals with Alzheimer's disease (AD) and Lewy body disease (LBD).^5^, ^6^ However, most sleep studies in dementia and mild cognitive impairment (MCI) utilize questionnaires of self‐reported or informant‐reported sleep.^7^ Similarly, although sleep disorder diagnoses are also associated with risk of developing neurodegenerative diseases,^2^ most studies looking at whether sleep disturbance increases risk of dementia have also typically used self‐report.^8^, ^9^

Self‐reported sleep measures are convenient and low burden, allowing for large‐scale studies. However, sleep assessed through self‐report instruments, such as the Pittsburgh Sleep Quality Index (PSQI),^10^ often does not align with objective sleep measures in older adults.^11^ While objective measures reliably demonstrate sleep disturbances in AD and LBD, studies utilizing subjective measures often provide conflicting results.^5^, ^12^ For example, a systematic review found worse sleep in individuals with MCI or AD in 93% of studies using objective measures, compared to only 41% using subjective measures.^13^ Population heterogeneity, methodology, and varying definitions of sleep parameters may partly explain these differences.^7^ However, patients with MCI or dementia also frequently experience impaired recall, working memory deficits, dyscalculia, or anosognosia, all of which could influence subjective reporting of sleep.^14^ In one study, memory impairment in patients with mild cognitive symptoms was shown to be biased toward better subjective sleep quality and explained paradoxical associations between poor sleep quality and more favorable AD‐related cerebrospinal fluid biomarkers and reduced hippocampal atrophy.^15^ Even in healthy adults, there is limited evidence that objective sleep parameters explain subjective sleep quality.^16^, ^17^ Therefore, subjective and objective sleep measures may be capturing overlapping but distinct concepts.

**RESEARCH IN CONTEXT**

1. **Systematic review**: A review of the literature found that sleep discrepancy has been reported in AD and dementia. However, alignment of estimated sleep parameters and how these might relate to distinct neurodegenerative diagnoses (e.g., AD vs LBD) has not been investigated.
2. **Interpretation**: Subjective appraisal of sleep differed markedly between disease cohorts. Participants with AD described satisfaction with sleep, whereas participants with LBD were dissatisfied with their sleep, while neither group objectively slept well. Discrepancies differentiated between cohorts with promising accuracy and highlight the need for multimodal sleep measurement.
3. **Future directions**: Measuring sleep in clinical trials and care will likely require objective measures for better accuracy alongside subjective measures. Larger‐scale studies are warranted to replicate and expand the diagnostic utility of subjective‐objective sleep discrepancy and to develop a subjective sleep measurement tool for this population to better align with objective measures.

Several studies in patients with MCI or dementia have identified a “mismatch” between subjective sleep quality and objective measures of sleep that are proxies for sleep quality, such as distribution of REM and non‐REM sleep or sleep efficiency (SE).^18^, ^19^, ^20^, ^21^ However, these studies largely did not compare the agreement or reliability of self‐reported sleep time variables compared to equivalent objective metrics, but rather whether poor subjective sleep quality is also seen in patients with poor objective sleep.

Discordance between subjective and objective measures of sleep is known as “subjective‐objective sleep discrepancy,” or, more simply, “sleep discrepancy.”^22^ Although sleep discrepancy could be interpreted as an “error” caused by inaccurate self‐report,^23^ self‐perceptions of sleep provide additional insight into sleep and plausibly even hold diagnostic or predictive utility. One study in cognitively healthy older adults showed that tau burden predicted worse objective sleep, while amyloid burden was associated with worse executive function and sleep underestimation.^24^ Another study in cognitively healthy older adults found that working memory deficits predicted sleep discrepancy in those with insomnia.^25^ Therefore, underlying pathology and clinical presentation may influence subjective and objective sleep parameters differentially, and if a pragmatic and reliable approach to collecting objective and subjective sleep parameters can be identified, examining both in memory clinic patients could contribute to differential diagnosis. Despite the high prevalence of sleep disturbance in memory clinic patients and its impact on cognition, currently sleep is not routinely assessed in memory clinics.^26^ Identifying the minimum amount of data needed to obtain reliable estimates of core sleep parameters may also encourage clinicians to consider routine assessment of sleep for secondary prevention in patients with MCI or improving quality of life in patients with dementia.^27^, ^28^

This study examined subjective and objective measures of core sleep parameters in adults with MCI or dementia due to AD or LBD, compared to age‐matched cognitively healthy adults. Specifically, the study assessed whether objective and subjective sleep measures differed across cohorts, the extent to which subjective and objective sleep parameters aligned, and whether sleep discrepancy could predict cohort membership.

## 2 METHODS

### 2.1 Overview

Remote Evaluation of Sleep To enhance understanding of Early Dementia (RESTED) was a three‐arm observational, longitudinal, prospective cohort study of individuals with MCI or early (mild to moderate) dementia due to AD or LBD and cognitively healthy age‐matched adults. A peer‐reviewed study protocol was published prior to the completion of data collection.^29^ Here, we focus specifically on subjective and objective measures of sleep collected during the baseline and main data collection period of the RESTED study. Results on feasibility,^30^ acceptability,^31^ and an autobiographical memory task used in the study^32^ have been published elsewhere. The study is reported in line with guidance from the Strengthening the Reporting of Observational Studies in Epidemiology (STROBE) statement.^33^

Each participant provided written informed consent prior to their baseline screening visit. This was followed by the main data collection phase comprising 8 weeks of wrist actigraphy supplemented with sleep diary responses. Participants additionally wore an electroencephalography (EEG) headband (Dreem 2)^34^ for 1 week.

### 2.2 Recruitment and study setting

Participants were recruited from multiple sources including the North Bristol National Health Service (NHS) Trust (NBT) Cognitive Disorders Clinic (a neurology‐led tertiary referral center for investigation and treatment of memory impairment), the NBT Movement Disorders clinic, a local research database of volunteers who had consented to be contacted about research opportunities, and the Join Dementia Research (JDR) database, a national dementia research registry.

### 2.3 Participant inclusion criteria

Full eligibility criteria are provided in Appendix S1. In summary, participants were older adults (≥50 years old) and required clinical diagnoses of AD or LBD according to standardized criteria^35^, ^36^, ^37^ or were age‐matched healthy controls without cognitive impairment. Those recruited to the AD or LBD cohorts were required to be at the MCI or mild dementia stages. Key exclusion criteria included clinically significant sleep disorders unrelated to AD or LBD pathology, severe medical or psychiatric comorbidities that may impact sleep, Montreal Cognitive Assessment (MoCA) score <11/30 at consent, and causes of dementia other than AD or LBD.

### 2.4 Data collection

#### 2.4.1 Demographics, medical screening, and history

Basic demographic information was collected, including age at consent, sex at birth, self‐reported ethnicity, and employment status. Data on medical diagnoses and medications were retrieved from participants and electronic medical records.

#### 2.4.2 Montreal cognitive assessment (MoCA)

Participants completed a MoCA with a trained researcher following consent before completing any other baseline assessments to confirm eligibility. The MoCA is a brief screening tool with high sensitivity and specificity for detecting MCI.^38^

#### 2.4.3 Neuropsychological assessment

Participants completed the Geriatric Depression Scale–Short Form (GDS‐15)^39^ and the Generalized Anxiety Disorder 7‐item questionnaire (GAD‐7)^40^ as self‐report measures of depression and anxiety, respectively.

#### 2.4.4 Plasma biomarkers of neurodegeneration

All participants were asked to undergo a blood test for the following biomarkers of neurodegeneration and AD: phosphorylated tau (p‐tau) 181, p‐tau217, neurofilament light chain (NFL), amyloid beta (Aß) 40, Aß42, and glial fibrillary acidic protein (GFAP).

#### 2.4.5 Sleep assessment

##### Sleep diary

Participants were asked to complete a daily sleep diary throughout the study period, consisting of the nine questions from the main Consensus Sleep Diary (CSD).^41^ Sleep diary entries were completed electronically using a software application named MyDignio, installed on a smart device (typically a smartphone or tablet). The use of the Dignio software streamlined sleep diary data collection and allowed the research team to remotely monitor completion of the diary and related assessments. Participants were prompted to complete their sleep diary each morning by the app.

##### Actigraphy (Axivity AX3)

Wrist actigraphy evaluated rest‐activity patterns during the day and macro‐architectural measures of sleep and wake at night including total sleep time (TST), SE, night‐time awakenings, and sleep (onset) latency (SL).^42^ Participants wore an Axivity AX3 wrist actigraph (three‐axis logging accelerometer) for the entire 8‐week study period.

Full configuration of sleep analysis is available in Appendix S2. Briefly, data were captured at a sampling rate of 25 Hz with raw Axivity AX3 files processed utilizing the open‐source GGIR Accelerometery Data Processing Software Package version 3.1‐2 in R.^43^ The package is capable of sleep detection with and without supporting sleep diary information relying on algorithms authored by van Hees et al.^43^, ^44^ Where available, sleep diary data were supplied to GGIR to improve sleep metric calculation performance, and when such data were absent, the “HDCZA” algorithm was utilized. Visual inspection was made on all nights to exclude all recordings during which the device was not worn.

##### EEG sleep recordings (Dreem 2)

Participants wore the Dreem 2 wireless multichannel EEG headband^34^ for seven consecutive nights during the 8‐week at‐home study period. Dreem 2 records, stores, and analyzes EEG data collected via five dry sensors: two frontal sensors at F7 and F8, one ground sensor at Fp2, and two occipital sensors at O1 and O2. Sleep recording was self‐initiated by the participant. Summary macro‐architectural characteristics of sleep were obtained from the proprietary Dreem 2 algorithm, which has been demonstrated to have comparable accuracy in sleep staging to polysomnography.^34^, ^45^

##### Sleep questionnaires

The primary instrument utilized for assessment of self‐report sleep was the PSQI, a 19‐item questionnaire assessing sleep quality and habitual sleep patterns over the preceding month. Its scoring is based on seven components: (i) subjective sleep quality, (ii) sleep latency, (iii) sleep duration, (iv) sleep efficiency, (v) sleep disturbances, (vi) use of sleep medicines, and (vii) daytime dysfunction. Each component is scored from 0 to 3, with higher scores indicative of worse sleep, yielding a global summated score (0 to 21). A total score above 5 is indicative of poor quality of sleep.^10^

Additional sleep questionnaires included the Epworth Sleepiness Scale (ESS) to assess excessive daytime somnolence,^46^ the STOP‐Bang Questionnaire to assess risk factors for obstructive sleep apnea (OSA),^47^ and a single screening question for REM sleep behavior disorder (RBD).^48^

##### Overnight pulse oximetry

Participants without a known Oxygen Desaturation Index (ODI) measurement within the last 6 months and who were not on continuous positive airway pressure (CPAP) treatment for diagnosed OSA were invited to complete two nights of overnight recording using a pulse oximeter (Nonin WristOx2 – Model 3150).

### 2.5 Statistical analysis

Data cleaning and analysis were performed using R Studio version 2024.04.2 utilizing R version 4.4.1 “Race For Your Life” statistical software. Basic descriptive statistics and demographics were reported by cohort for relevant outcomes with appropriate tests for group comparison depending on data normality assessed visually and using the Shapiro–Wilkes Test. Objective sleep outcome measures as recorded by actigraphy and EEG are presented in summary descriptive form alongside corresponding values derived from the PSQI. Regression models determining cohort differences in objective and subjectively derived sleep metrics throughout the manuscript were checked for adherence to standard assumptions including multicollinearity. Adjusted models control for the following potential confounders of age, sex at birth, affective symptoms (GAD‐7 total score, GDS‐15 total score), sedative medication use, and suspected untreated OSA (defined as ODI ≥ 15 during at least one night of pulse oximetry or, where unavailable, STOP‐Bang ≥ 5 and not on CPAP or known OSA and declined treatment).

Missing data were handled using a complete‐case approach. No imputation was performed with the sole exception of specific sleep diary values in circumstances described in Appendix S3.

Parameter effect sizes (Cohen's *d*) are reported for parameters compared across cohort. In view of small sample sizes, the absence of a statistically significant difference should be interpreted with caution (see Appendix S3 for indicative Minimum Detectable Effect calculation).

The subjective sleep quality outcome measure is presented in descriptive form with additional analyses performed to determine whether differences in cohort remain after correction for confounders in an adjusted ordinal regression model using the “clm” function of the ordinal package in R. Objective sleep parameters as predictors of subjective self‐reported sleep quality were evaluated in a similar model including interaction terms to encompass cohort membership and a final model also adjusting for the previously mentioned confounders.

#### 2.5.1 Night‐by‐night bias and precision in self‐reported sleep by CSD versus EEG (episodic sleep discrepancy)

To calculate mean error and precision of estimates on a night‐by‐night basis comparing CSD metrics provided the following day after the previous night EEG recording and whether these differed by cohort, a linear mixed effects location‐scale model from the brms package in R was utilized providing estimates of mean error and residual standard deviation (sigma) by cohort with intercept allowed to vary by participant.

#### 2.5.2 Accuracy of self‐reported sleep by PSQI compared to objective measurement (habitual sleep discrepancy)

The PSQI instructs participants to provide information relating to their usual sleep across the past month, giving a habitual measure of subjective sleep. For objective comparison, data must therefore be aggregated and averaged across several nights of recording.^22^ To determine which of our objective measures would provide the most meaningful comparison for the PSQI (prolonged actigraphy collection or shorter EEG collection), we calculated the minimum number of nights required for each modality to produce a stable participant mean across four core sleep parameters: TST, SL, SE, and WASO. This was calculated by use of the Spearman Brown correction method relying on intraclass correlation coefficients. The modality providing the most stable mean estimates was then compared to the corresponding PSQI self‐reported estimate to determine habitual sleep discrepancy. The extent of this discrepancy was then analyzed descriptively by cohort before a linear regression model was specified with confounders and dummy variables indicating cohort membership. A further regression modeled associations between the degree of discrepancy and plasma biomarkers, agnostic to cohort membership.

#### 2.5.3 Predictive capacity of subjective and objective metrics to determine cohort membership

The capacity of discrepancies with larger intercohort effect sizes to predict cohort membership was assessed through the use of a multinomial regression model with the nnet package in R. A stepwise approach was used whereby discrepancy parameters were added to the model alongside PSQI subjective sleep quality score. The model achieving the best overall accuracy was then selected.

Receiver operator characteristic (ROC) curves were produced utilizing the pROC package in R to assess the accuracy of these metrics in different combinations of study population.

## 3 RESULTS

### 3.1 Baseline participant characteristics

Forty participants (AD *n* = 10, LBD *n* = 10, and control *n* = 20) were included in the analysis. Further details on recruitment and retention are published in our feasibility paper.^30^ Baseline participant characteristics are summarized in Table 1. There was no evidence to support differences between cohorts for age, sex, education, ethnicity, use of sleep medication, or anxiety score. A higher mean (standard deviation [SD]) GDS‐15 score was seen in the AD and LBD cohorts with moderate to large effect size, and these values were indicative of mild depressive symptomatology only.

**TABLE 1:** RESTED baseline characteristics.

Variable	AD cohort (*n* = 10)	LBD cohort (*n* = 10)	Control group (*n* = 20)	AD versus control *p* value	LBD versus control *p* value	AD versus LBD *p* value
	Mean (SD) Count (%)	Mean (SD) Count (%)	Mean (SD) Count (%)			
**Demographic**						
Age	69.2 (7.9)	73.9 (2.8)	70.3 (5.7)	0.713^a^	0.026^a^	0.102^a^
Female sex	2 (20)	2 (20)	5 (25)	1^b^	1^b^	1^b^
Ethnicity						
White–British	10 (100)	10 (100)	20 (100)	1^b^	1^b^	1^b^
>12 years of education	6 (60)	5 (50)	16 (80)	0.465^b^	0.205^b^	1^b^
Shift working						
Current	0 (0)	0 (0)	2 (10)	0.796^b^	0.796^b^	–
Historic	7 (70)	5 (50)	11 (55)	0.693^b^	1^b^	0.648^b^
**OSA screening**						
STOP‐Bang ≥ 5	1 (10)	4 (40)	2 (10)	1^b^	0.146^b^	0.302^b^
Possible Untreated Moderate‐Severe OSA	1 (10)	3 (30)	5 (25)	0.628^b^	1^b^	0.576^b^
**Behavioral data**						
Use of sleep medication	1 (10)	4 (40)	1 (5)	1^b^	0.057^b^	0.302^b^
Epworth Sleepiness Scale	8.3 (4.9)	8.1 (4.2)	5.7 (4.8)	0.139	0.107	1
RBD single question screen	2 (20)	5 (50)	1 (5)	0.519^b^	0.015^b^	0.348^b^
**Cognitive/psychological data**						
MoCA	22.7 (4.9)	21.4 (4.5)	26.9 (1.6)	0.010	<0.001	0.425
GDS‐15 total	4.5 (3.5)	4.1 (2.3)	2.2 (2.2)	0.034	0.055	1
GAD‐7 total	5.6 (5.2)	3.5 (2.1)	3.5 (4.3)	0.161	0.424	0.761
**Plasma biomarkers** ^c^	*n* = 9	*n* = 9	*n* = 15			
Aβ 42	6.35 (1.3)	6.76 (1.3)	7.06 (1.2)	0.263	0.558	0.666
P‐tau 181	26.23 (7.1)	24.88 (8.8)	24.86 (12.7)^d^	0.253	0.705	0.666
P‐tau 217	0.701 (0.4)^e^	0.75 (0.4)	0.557 (0.4)	0.076	0.129	1
Aβ 42/40 ratio	0.064 (0)	0.062 (0)	0.072 (0)	0.012	0.003	0.489
P‐tau 181/ Aβ 42 ratio	4.336 (1.6)	4.055 (1.6)^f^	3.657 (1.5)	0.215	0.506	0.606
GFAP	102.2 (28)	140.9 (56)^f^	103.5 (48)	0.815	0.108	0.297

*Note*: *p* values calculated utilizing Mann–Whitney U test unless otherwise specified.

Abbreviations: Aβ, amyloid beta; AD, Alzheimer's disease; GAD‐7, Generalized Anxiety Disorder–7 Scale; GDS‐15, Geriatric Depression Scale (15‐item); GFAP, glial fibrillary acidic protein; LBD, Lewy body disease; MoCA, Montreal Cognitive Assessment; OSA, obstructive sleep apnea; p‐tau, phosphorylated tau; RBD, REM Sleep Behavior Disorder; SD, standard deviation.

^a^ *p* value calculated using independent samples *t*‐test.

^b^ *p* value calculated using chi‐squared test.

^c^ Plasma biomarker data available for AD *n* = 9; control *n* = 15, unless otherwise stated.

^d^ p‐tau181 results available for control *n* = 18.

^e^ p‐tau217 results available for AD *n* = 8.

^f^ p‐tau181/Aβ 42 ratio results available for LBD *n* = 8.

### 3.2 Objective and subjective sleep parameters by cohort

A total of 2134 nights of actigraphy data were analyzed across the full cohort. Mean (SD) actigraphy recordings per participant were 53.4 (8.0). A total of 209 nights of EEG data were utilized for analysis with a mean (SD) of 5.5 (1.8) recordings per participant. For further information relating to actigraphy and EEG data quality and handling, see Appendix S3.

#### 3.2.1 Trends were identified toward less favorable objectively derived sleep parameters in individuals with AD and LBD, but few reached statistical significance

AD sleep duration (TST) as defined by actigraphy was shorter in the AD cohort versus control by approximately 47 min (Cohen's *d* [95% CI] = −0.873 [−1.7, −0.05], *p* = 0.036) and SL was prolonged by approximately 7 min (Cohen's *d* [95% CI] = 0.925 [0.10, 1.8], *p* = 0.028). Otherwise, there were general trends toward objective measures of sleep in the AD and LBD cohorts reflecting a less favorable sleep pattern (Table 2).

**TABLE 2:** Mean objective and subjective estimation of quantitative and qualitative sleep parameters by cohort.

Modality	AD mean (SD)	LBD mean (SD)	Control mean (SD)	AD versus controls Cohen's *d* (95% CI)	AD versus Cont *p*	LBD versus controls Cohen's *d* (95% CI)	LBD versus control *p*	AD versus LBD Cohen's *d* (95% CI)	AD versus LBD *p* value
**Quantitative parameter**									
Total sleep time (TST), min									
Act	332 (53)	355 (66)	379 (54)	−0.873 (−1.7, −0.05)	0.036	−0.418 (−1.2, 0.38)	0.329	−0.380 (−1.3, 0.57)	0.407
EEG	393 (43)	432 (49)	405 (32)	−0.332 (−1.1, 0.47)	0.453	0.706 (−0.15, 1.6)	0.161	−0.841 (−1.9, 0.17)	0.088
PSQI	447 (48)	459 (96)	406 (67)	0.667 (−0.15, 1.5)	0.067	0.685 (−0.13, 1.5)	0.139	−0.158 (−1.1, 0.78)	0.730
Sleep latency (SL), min									
Act	24.2 (7.4)	21.1 (4.6)	17.4 (7.3)	0.925 (0.10, 1.8)	0.028	0.562 (−0.24, 1.4)	0.103	0.505 (−0.45, 1.5)	0.277
EEG	25.5 (14)	26.4 (14)	17.6 (14)	0.558 (−0.26, 1.4)	0.168	0.624 (−0.23, 1.5)	0.139	−0.065 (−1, 0.9)	0.888
PSQI	7.2 (4.5)	25 (35)	18.5 (21)	−0.660 (−1.5, 0.15)	0.028	0.251 (−0.55, 1)	0.594	−0.721 (−1.7, 0.25)	0.140
Sleep efficiency (SE), %									
Act	75 (10)	70.9 (14)	79.3 (7.3)	−0.510 (−1.3, 0.3)	0.262	−0.858 (−1.7, −0.03)	0.093	0.338 (−0.61, 1.3)	0.461
EEG	83 (5.7)	79.6 (6.8)	83.4 (6.7)	−0.063 (−0.87, 0.74)	0.866	−0.568 (−1.4, 0.28)	0.181	0.548 (−0.44, 1.5)	0.255
PSQI	81.8 (10)	80.3 (15)	75.9 (16)	0.401 (−0.4, 1.2)	0.237	0.271 (−0.53, 1.1)	0.483	0.117 (−0.82, 1.1)	0.796
Wake after sleep onset (WASO), min									
Act	65.7 (35)	104 (65)	60.7 (36)	0.140 (−0.65, 0.93)	0.720	0.922 (0.09, 1.8)	0.073	−0.737 (−1.7, 0.23)	0.122
EEG	47.7 (25)	80.5 (40)	57.4 (28)	−0.357 (−1.2, 0.45)	0.353	0.710 (−0.14, 1.6)	0.149	−0.989 (−2, 0.038)	0.056
PSQI	99.3 (65)	88 (66)	120 (106)	−0.224 (−1, 0.57)	0.504	−0.343 (−1.1, 0.46)	0.312	0.172 (−0.77, 1.1)	0.705
Time in bed (TIB), min									
Act	445 (50)	504 (67)	478 (47)	−0.699 (−1.5, 0.12)	0.095	0.476 (−0.33, 1.3)	0.295	−1.000 (−2, −0.01)	0.039
EEG	474 (47)	547 (71)	489 (50)	−0.304 (−1.1, 0.5)	0.434	1.01 (0.14, 1.9)	0.047	−1.240 (−2.3, −0.18)	0.020
PSQI	554 (88)	572 (58)	545 (76)	0.109 (−0.68, 0.9)	0.792	0.381 (−0.42, 1.2)	0.293	−0.241 (−1.2, 0.7)	0.597
**Qualitative parameter**									
PSQI total	3.9 (1.9)	8.2 (3.6)	5.75 (3.9)	−0.543 (−1.3, 0.26)	0.093	0.641 (−0.17, 1.5)	0.105	−1.49 (−2.5, −0.43)	0.005
PSQI subjective sleep quality subcomponent	0.5 (0.53)	1.4 (0.7)	1 (0.86)	−0.651 (−1.5, 0.16)	0.060	0.493 (−0.31, 1.3)	0.186	−1.45 (−2.5, −0.4)	0.005
Self‐report satisfied with sleep *n* (%)	8 (80)	5 (50)	14 (70)	OR = 1.63 (0.28, 15)	0.884	OR = 0.444 (0.085, 2.2)	0.503	OR = 10.4 (1.8, 96)	0.013^a^

*Note*: *p* values are unpaired *t*‐tests unless otherwise stated.

Abbreviations: Act, actigraphy; AD, Alzheimer's disease; CI, confidence interval; EEG, electroencephalography; LBD, Lewy body disease; OR, odds ratio; PSQI, Pittsburgh Sleep Quality Index.

^a^ Chi‐squared test.

#### 3.2.2 Subjective estimation of these core sleep parameters was more favorable in the AD and LBD cohorts compared to controls

The AD and LBD cohorts on average estimated longer TST, higher SE, and lower WASO compared to control participants (Table 2). The AD cohort also estimated lower SL than the control cohort by 11 min (Cohen's *d* [95% CI] = −0.660 [−1.5, 0.15], *p* = 0.028).

#### 3.2.3 Subjective sleep quality differed between AD and LBD cohorts

Subjective appraisal of sleep according to the PSQI total score and the subjective sleep quality subcomponent generally followed a ranking of LBD, control, and then AD cohorts representing least to most sleep satisfaction (Table 2). Differences in appraisal between AD and LBD (at the polar ends of this distribution) were of large effect size and statistically significant.

To determine whether these differences between cohort remained after correction for confounders, an ordinal regression model was created controlling for confounders as specified in Section 2. AD cohort membership was associated with better subjective self‐report (i.e., lower PSQI values), OR [95% CI] = 0.047 [0.005, 0.419], with a trend toward worse subjective self‐report in the LBD cohort; OR [95% CI] = 4.37 [0.73, 26.0]. Further details are provided in the Supporting Information.

#### 3.2.4 Objective sleep efficiency predicted night‐by‐night subjective sleep quality in all cohorts. However, objective sleep efficiency predicted habitual subjective sleep quality only in healthy older adults

To determine which objective measures of sleep best predicted subjective sleep quality, two analyses were performed. First, EEG data were matched with nightly diarized reports from the CSD of subjective sleep quality on a 5‐point ordinal scale ranging from “very poor” to “very good.” In the adjusted model, higher EEG sleep efficiency was strongly associated with better subjective sleep quality across cohorts. Each standard deviation increase in SE corresponded to a 3.74‐fold increase in the odds of reporting a higher sleep quality category (SE OR [95% CI] = 3.74 [2.00, 6.99]). Longer TST was also unexpectedly associated with reduced odds of higher sleep quality categories, but only in the LBD cohort (LBD TST OR [95% CI] = 0.327 [0.14, 0.74])[Fig alz71377-fig-0001].

A second model analyzing longer‐term “average estimates” utilized mean actigraphy‐derived objective parameters as predictors of the PSQI subjective sleep quality score designed to reflect appraisal over 1 month. Here, a crude ordinal regression model found associations between higher SE and better subjective sleep quality in the control cohort (SE Est = −2.49, OR [95% CI] = 0.083 [0.009, 0.777]), but not in the AD or LBD cohort. No associations maintained statistical significance in adjusted models. For full results for both models, see Appendix S4.

### 3.3 Sleep discrepancy by cohort

#### 3.3.1 Compared to EEG, participants with AD reported shorter sleep latency in sleep diaries for the corresponding night. There was also supportive evidence for greater variability/reduced reliability of estimates of sleep duration and sleep efficiency in participants with AD and LBD compared to controls

**FIGURE 1 alz71377-fig-0001:**
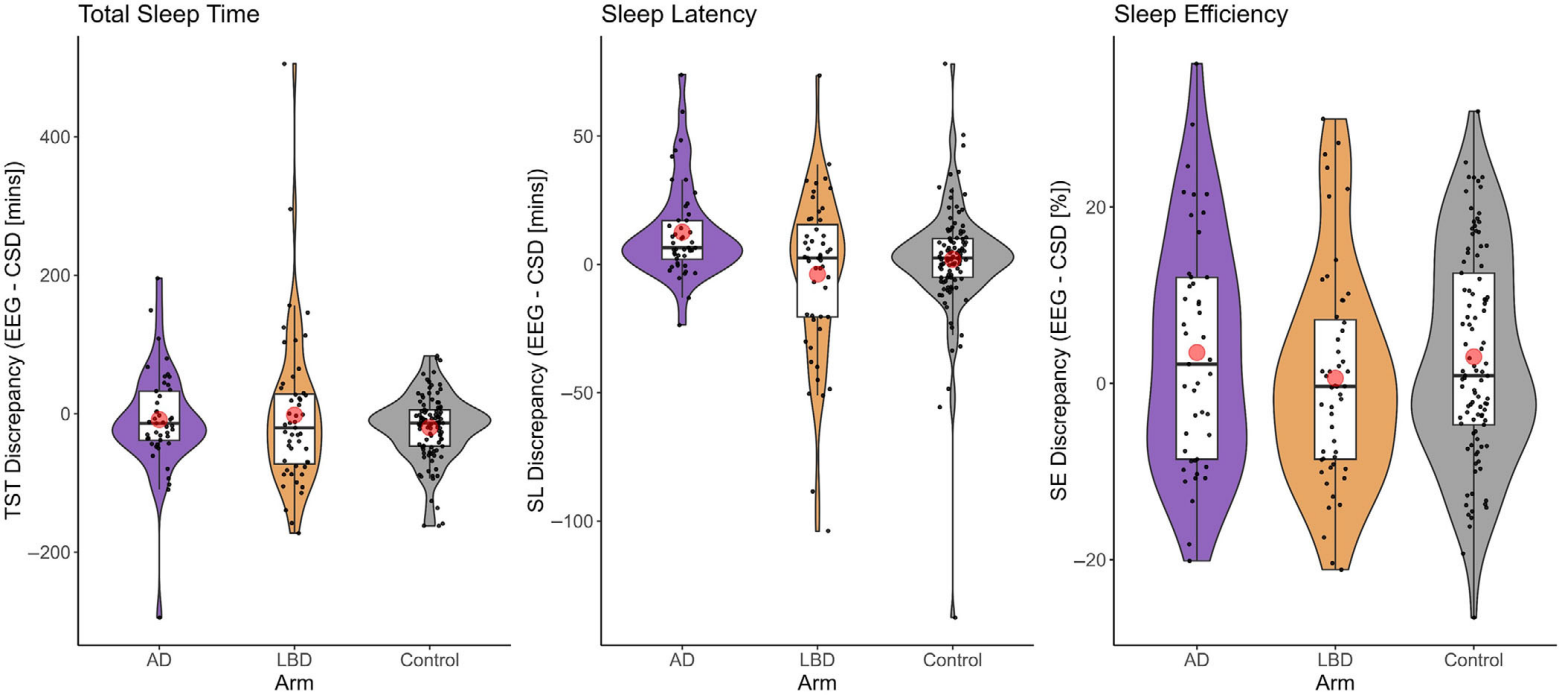
Discrepancy between nightly CSD versus EEG estimates of TST, SL, and SE by Cohort. Discrepancies between episodic (nightly) estimates of TST, SL, and SE as obtained by CSD (sleep diaries) versus corresponding Dreem 2 (objective EEG) measurement by cohort, AD (purple), LBD (orange), control (gray). Mean discrepancy (red dot) is similarly demonstrated by cohort. AD, Alzheimer's disease; CSD, Consensus Sleep Diary; EEG, electroencephalography; LBD, Lewy body disease; SE, sleep efficiency; SL, sleep (onset) latency; TST, total sleep time.

On a nightly basis, we compared EEG‐derived metrics to equivalent metrics derived from sleep diaries for the corresponding night to calculate episodic sleep discrepancy (Figure 1). Estimates of bias (mean [lower 95% CI, upper 95% CI]) and reliability (sigma, *σ*) were calculated. Healthy older adults self‐reported longer TST than EEG‐derived estimates by 21.0 [0.54, 42.49] minutes. Estimates were less reliable (reflected by higher sigma, *σ*) in AD and LBD cohorts compared to control, AD = 0.55 [0.28, 0.83], LBD = 0.97 [0.71, 1.25]. Participants with AD self‐reported shorter SL than EEG‐derived estimates, mean = 13.1 [2.57, 24.4] minutes with higher and lower reliability in AD and LBD cohorts respectively compared to control, mean *σ* AD = −0.28 [−0.54, −0.003], mean *σ* LBD = 0.28 [0.02, 0.56]. There was no systematic bias demonstrated in estimates of SE but further evidence for lower reliability, mean *σ* AD = 0.32 [0.05, 0.6], mean *σ* LBD = 0.35 [0.09, 0.61]. For full results see Appendix S4.

#### 3.3.2 Multiple PSQI sleep parameters reported by individuals with AD and LBD indicated lower sleep disturbance compared to actigraphy

To compare estimates of “usual” or habitual sleep obtained utilizing the PSQI, we first determined which method of objective recording (EEG over 7 days or actigraphy over 8 weeks) provided the most reliable, stable mean.

**FIGURE 2:**
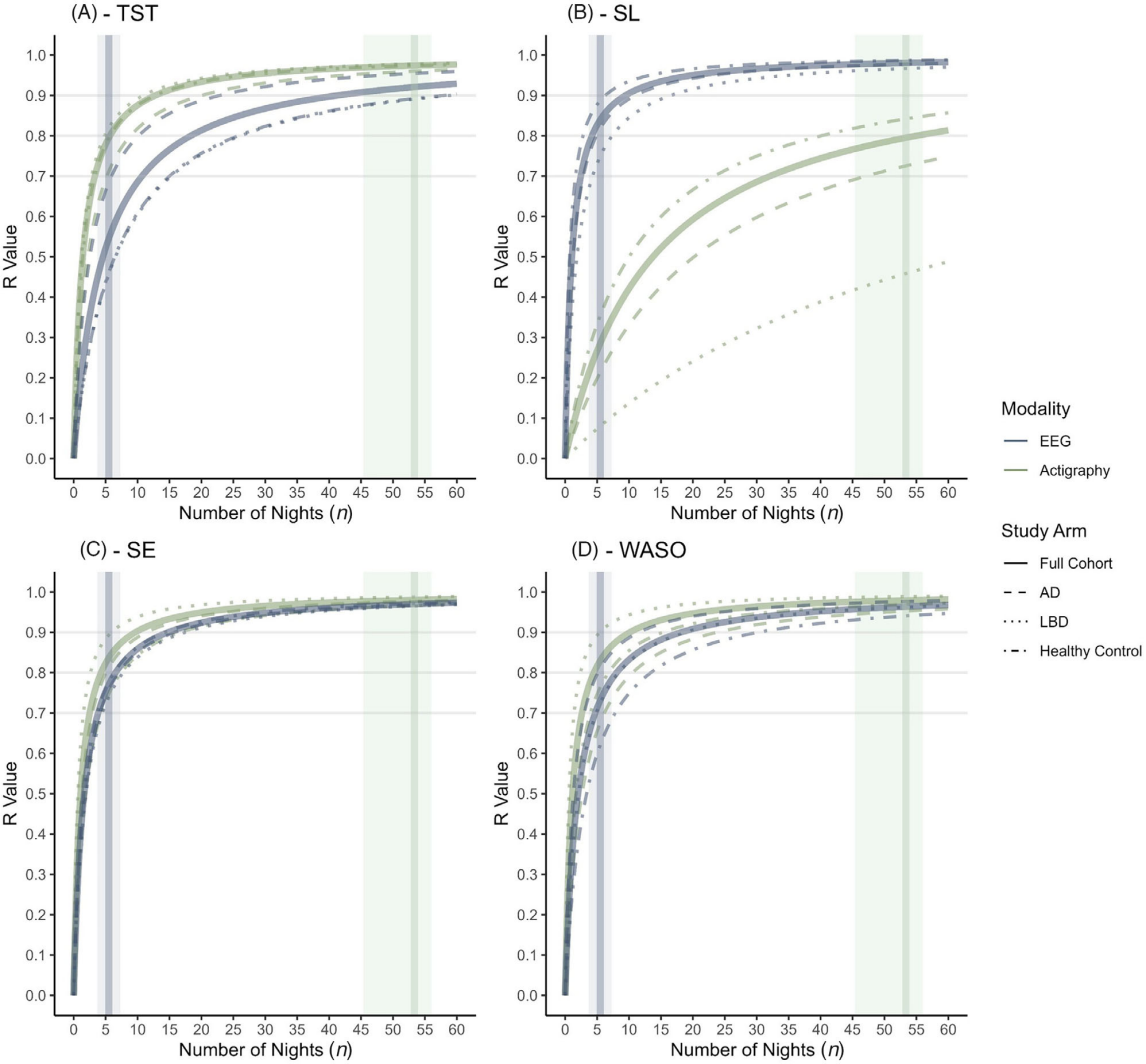
Relationship between number of recordings and stability of sleep parameter mean by modality and cohort. The reliability/stability of the participant mean (*r*) is plotted against the number of nights of measurement for TST (A), SL (B), SE (C), and WASO (D) for EEG (blue lines) and actigraphy (green lines). The full cohort (thick solid line) is compared with the stratified arms; control (dot‐dash), AD (dashed), and LBD (dotted). The mean (vertical line) and standard deviation (shaded area) for collected nights per participant in the RESTED study is shown for actigraphy (green) and EEG (blue). Acceptable (*r* = 0.7), good (*r* = 0.8), and excellent (*r* = 0.9) cut points for R are highlighted by horizontal dashed lines. AD, Alzheimer's disease; EEG, electroencephalography; LBD, Lewy body disease; SE, sleep efficiency; SL, sleep (onset) latency; TST, total sleep time; WASO, wake after sleep onset.

A mean of 53.4 nights of actigraphy and 5.5 nights of EEG were recorded per participant. With 53.4 nights of actigraphy “excellent” mean reliability was provided for TST (*r* = 0.974), WASO (*r* = 0.979), and SE (*r* = 0.980), and “acceptable” reliability was provided for SL (*r* = 0.795). With 5.5 nights of EEG recording good reliability was provided for SL (*r* = 0.840) but poor reliability for TST (*r* = 0.545) and only acceptable reliability for SE (*r* = 0.770) and WASO (*r* = 0.732) (Figure 2 and Appendix S4). To calculate habitual sleep discrepancy between self‐reported sleep encompassing a 4‐week period (the PSQI) and an equivalent reliable average derived from objective measurements, actigraphy‐derived means were therefore utilized.

**FIGURE 3:**
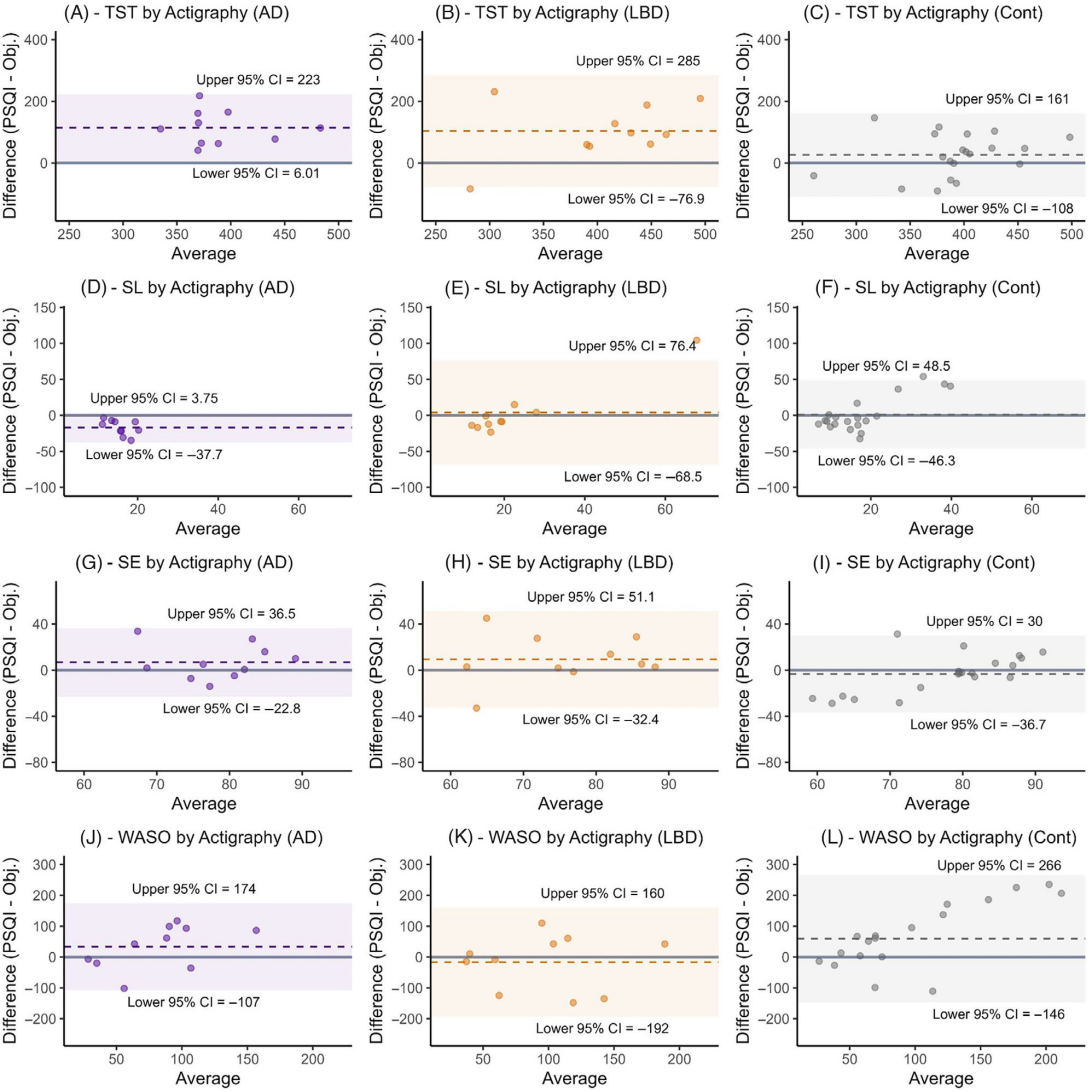
Bland–Altman charts of PSQI versus actigraphy by cohort. Bland–Altman charts by cohort (left [purple] – AD, central [orange] – LBD, right [gray] – control). Mean difference values represented by dashed line and 95% confidence intervals by shaded areas. Obj., objective measure (actigraphy); SE, sleep efficiency; SL, sleep (onset) latency; TST, total sleep time; WASO, wake after sleep onset.

The mean differences by cohort between PSQI and actigraphy‐derived objective measures of TST, SE, and SL are shown in Table 3 with Bland–Altman charts in Figure 3. For EEG‐derived metrics demonstrating similar patterns, see Appendix S4.

**TABLE 3:** Mean actigraphy versus PSQI discrepancy by cohort.

Sleep discrepancy	AD mean (SD)	LBD mean (SD)	Control mean (SD)	AD versus controls Cohen's *d* (95% CI)	AD versus controls *p* ^*^	LBD versus controls Cohen's *d* (95% CI)	LBD versus controls *p* ^*^	AD versus LBD Cohen's *d* (95% CI)	AD versus LBD *p* ^*^
TST discrepancy (min)	115 (55)	104 (92)	26.4 (69)	1.360 (0.49, 2.2)	0.001	1.000 (0.17, 1.8)	0.034	0.140 (−0.8, 1.1)	0.758
SL discrepancy (min)	−17.0 (11)	3.93 (37)	1.12 (24)	−0.870 (−1.7, −0.04)	0.008	0.097 (−0.7, 0.89)	0.831	−0.769 (−1.7, 0.21)	0.115
SE discrepancy (%)	6.84 (15)	9.39 (21)	−3.35 (17)	0.620 (−0.19, 1.4)	0.111	0.689 (−0.13, 1.5)	0.121	−0.138 (−1.1, 0.8)	0.762
WASO discrepancy (min)	33.6 (72)	−16.4 (90)	59.8 (105)	−0.273 (−1.1, 0.52)	0.430	−0.758 (−1.6, 0.06)	0.052	0.616 (−0.35, 1.6)	0.186

*Note*: TST, SL, SE, and WASO discrepancy calculated by subtracting actigraphy‐derived parameters from equivalent metrics obtained from self‐report (PSQI) metrics.

Abbreviations: AD, Alzheimer's disease; CI, confidence interval; LBD, Lewy body disease; PSQI, Pittsburgh Sleep Quality Index; SE, sleep efficiency; SL, sleep (onset) latency; TST, total sleep time; WASO, wake after sleep onset.

* *p*‐values calculated by independent samples *t*‐test.

Individuals within the AD and LBD cohorts reported longer TST by approximately 115 and 104 min, respectively, compared to actigraphy. The AD cohort also reported a SL that was shorter by 17 min. There were trends in both cohorts toward higher self‐reported SE. SE was longer by mean 6.8% in the AD cohort and 9.4% in the LBD cohort, but lower in the control cohort by 3.4%. These differences did not reach predefined statistical significance but were of moderate effect size.

Bland–Altman charts show these tendencies to be persistent across the range of sleep durations in the AD and LBD cohorts and across the range of SL in the AD cohort (Figure 3). Within the control cohort, individuals with less favorable SE and SL appeared more likely to estimate even lower SE and even more prolonged SL subjectively. In other words, objective impairment in these parameters was associated with overestimation of their severity.

#### 3.3.3 Lower self‐reported sleep disturbance for specific sleep parameters and differences in subjective appraisal of sleep quality in the AD and LBD cohorts persisted after correction for confounders

Given intercohort differences in subjective‐objective sleep assessment, linear regression models were used to adjust for potential confounders with crude and adjusted models summarized in Appendix S4.

Adjusted models showed that, holding other variables constant, sleep duration (TST) was biased toward overestimation by 120 min, *p* < 0.001 (AD cohort) and 62 min, *p* = 0.040 (LBD cohort) compared to control. Trends toward an overestimation bias for SE were also identified of approximately 15.0%, *p* = 0.066 (AD cohort) and 10.1%, *p* = 0.194 (LBD cohort) compared to control. A bias toward underestimation of *log* sleep latency was also shown in the AD cohort, approximately 75.6% of the control value.

**FIGURE 4:**
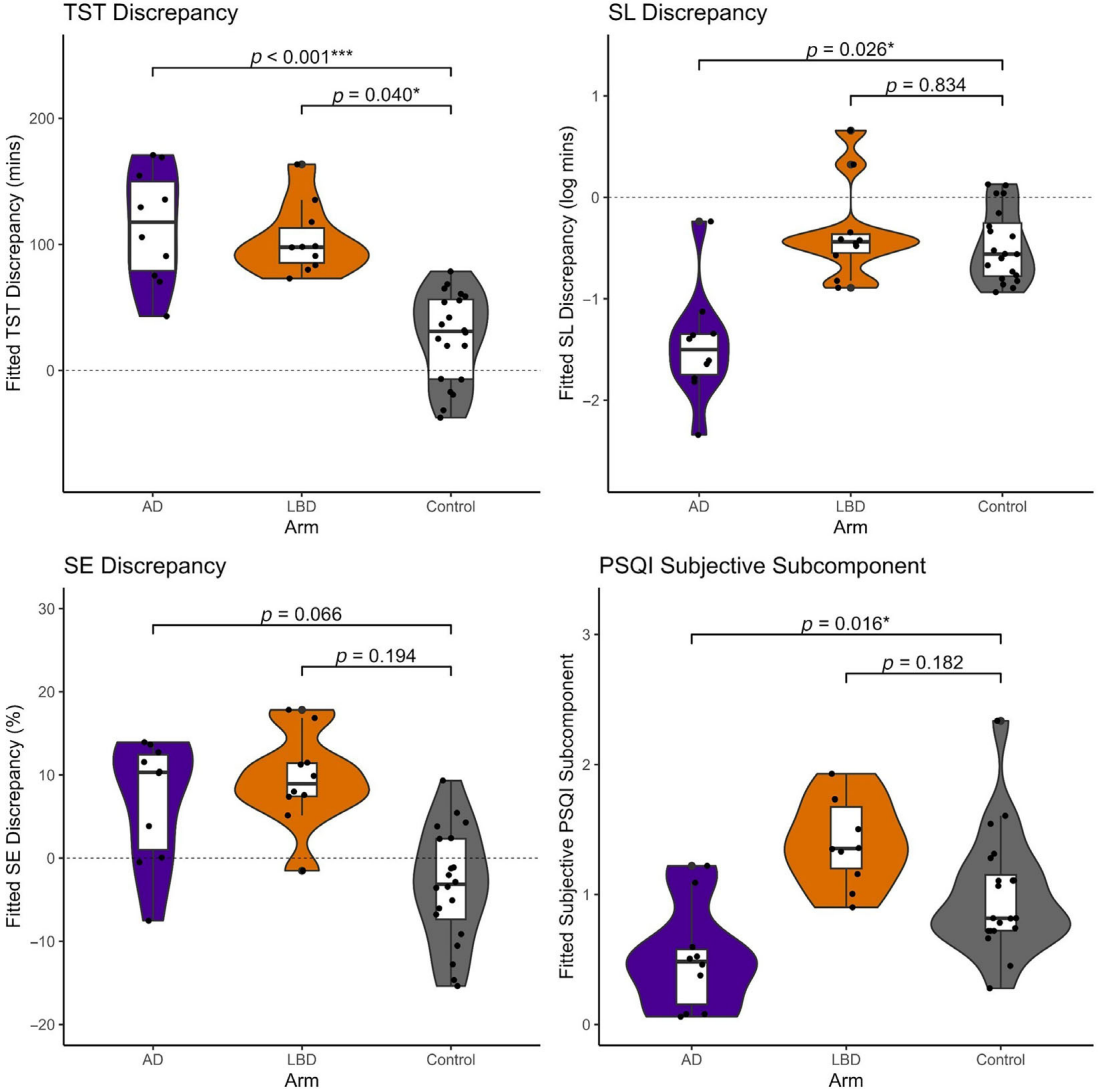
Fitted values for key parameters differentiating cohort. Fitted linear regression values for habitual sleep discrepancies in TST, SL, and SE and subjective estimation of sleep by cohort. AD, Alzheimer's disease; LBD, Lewy body disease; PSQI, Pittsburgh Sleep Quality Index; SL, sleep (onset) latency; SE, sleep efficiency; TST, total sleep time.

Key parameters demonstrating differences between cohorts are shown in Figure 4.

#### 3.3.4 Trends were supportive of associations between degree of sleep discrepancy and plasma neurodegenerative biomarkers, but these did not survive correction for multiple comparisons

The relationships between habitual sleep discrepancy and plasma neurodegenerative biomarkers were also assessed with linear regression models specified with the biomarker as a dependent variable. Trends were supportive of the associations found above with each standard deviation reduction in the Aβ 42/40 ratio, an increase in ptau‐217, and an increase in NFL associated with bias toward reporting longer TST by approximately 28, 35, and 32 min, respectively, in the adjusted models. Each standard deviation increase in NFL was further associated with an approximately 39% bias toward reporting shorter habitual SL. However, associations did not survive correction for multiple comparisons utilizing the false discovery rate. For full results see Appendix S4.

### 3.4 Predictive capacity of subjective and objective sleep characteristics to determine cohort membership

#### 3.4.1 Use of habitual sleep discrepancy and subjective sleep quality achieved high accuracy in predicting cohort membership

Given the associations observed, we then determined the degree to which combinations of variables could predict cohort membership utilizing the multinomial regression model specified in Section 2. The model producing optimal performance was specified as follows:

$$\begin{array}{ccc} \mathrm{Cohort}∼a & + & \beta 1*\mathrm{tst}\_\mathrm{disc}+\beta 2*\mathrm{lat}\_\mathrm{disc}+\beta 3*\mathrm{eff}\_\mathrm{disc}\\ & + & \beta 4*\mathrm{psqi}\_\mathrm{subjective} \end{array}$$

where AD = dummy variable expressing AD cohort membership, tst_disc = (PSQI sleep duration − mean actigraphy sleep duration), lat_disc = (log(PSQI sleep latency) − log (mean actigraphy sleep latency), eff_disc = (PSQI raw sleep efficiency − mean actigraphy sleep efficiency), psqi_subjective − subjective subcomponent of PSQI.

This model resulted in the following classifications: AD cohort (AD, *n* = 7; LBD, *n* = 0; control, *n* = 3), LBD cohort (AD, *n* = 1; LBD, *n* = 6; control, *n* = 3), control cohort (AD, *n* = 1; LBD, *n* = 1; control, *n* = 18). This yielded sensitivity (AD = 0.70, LBD = 0.60, control = 0.90), specificity (AD = 0.93, LBD = 0.97, control = 0.70), and balanced accuracy (AD = 0.82, LBD = 0.78, control = 0.80). For full predictive model statistics see Appendix S4.

**FIGURE 5:**
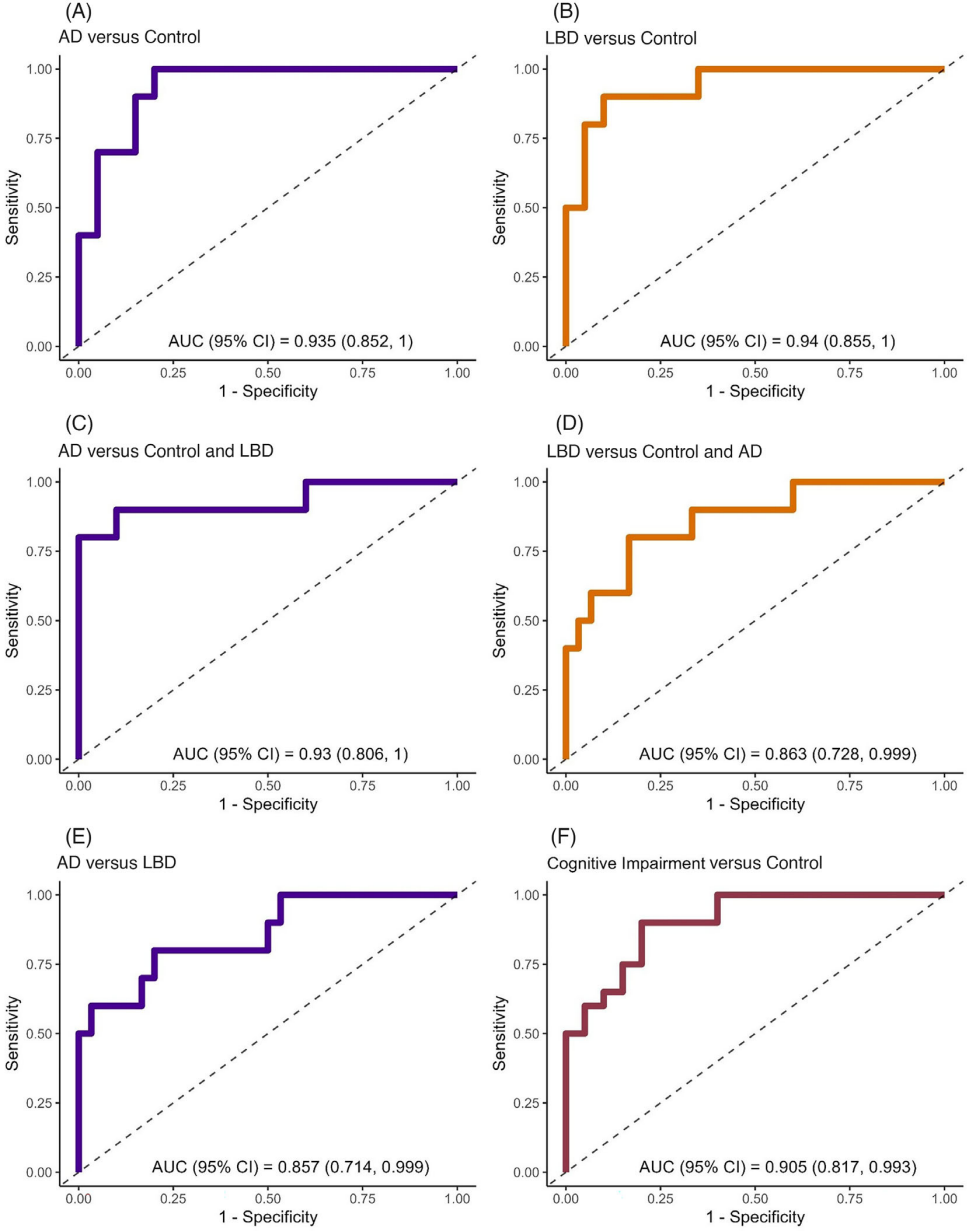
ROC curve for specified model predicting cohort membership. ROC of regression model predictors in correctly classifying participants by cohort utilizing different cutoffs (R). Potential levels shown in light green (specificity 80%, sensitivity 100%) and dark red (specificity 85% and sensitivity 90%). AD, Alzheimer's disease; AUC, area under the curve; LBD, Lewy body disease; ROC, receiver operator characteristic.

ROC curves are shown in Figure 5. Good to excellent degrees of accuracy were obtained with area under the curve (AUC) estimates ranging from 0.857 (95% CI [0.714, 0.999]) to 0.940 (95% CI [0.855, 0.999]) depending on the population comparison.

Finally, we analyzed the misclassified group to determine whether there were differences in demographics, sleep, or neuropsychological characteristics compared to the correctly categorized group. No evidence was found to support differences in objective sleep parameters. However, there were trends toward higher self‐reported depression and anxiety, as well as worse subjective sleep, in the misclassified group. Mean (SD) full cohort GDS‐15 total misclassified = 5.22 (3.3), correctly classified = 2.68 (2.4), Cohen's *d* (95% CI) = 0.98 (0.18, 1.8), *p* = 0.058. Mean (SD) full cohort GAD‐7 total misclassified = 7.11 (5.6), correctly classified = 3.16 (3.2), Cohen's *d* (95% CI) = 1.03 (0.23, 1.8), *p* = 0.074. Mean (SD) full cohort PSQI total misclassified = 7.44 (5.1), correctly classified = 5.45 (3.2), Cohen's *d* (95% CI) = 0.545 (−0.23, 1.3), *p* = 0.295. Further models revealed these trends to be evident in all three cohorts (see Appendix S4 for full results).

## 4 DISCUSSION

While poor sleep is increasingly recognized as a symptom and risk factor of dementia,^49^ defining good quality sleep and optimally measuring sleep remains challenging, particularly in real‐world settings.^50^ To date, sleep studies in MCI and dementia have mostly used questionnaires to measure sleep.^7^ The present study examined subjective and objective sleep and how closely sleep parameters aligned in participants with MCI and dementia due to AD and LBD. Here, we show that participants with AD and LBD reported better subjective sleep parameters (in the PSQI) than objectively recorded (by actigraphy). Subjective sleep quality and sleep discrepancy for three core sleep parameters (TST, SL, SE) discriminated between healthy older adults and participants with AD and LBD with good to excellent degrees of accuracy, highlighting potential clinical utility. Interestingly, positive sleep discrepancy was reliably observed when assessing habitual or average sleep over several weeks, but not on a night‐by‐night basis.

Despite typically self‐reporting sleep duration within the optimal range of 7 to 8 hours,^51^ on average participants with AD and LBD had around 6 hours of objectively recorded sleep per night. Participants with AD reported general satisfaction with their sleep, and even though participants with LBD were dissatisfied with their sleep, they reported lower sleep disturbance than recorded objectively. Relying on sleep parameters collected via patient self‐report in memory clinics or research, particularly habitual sleep questionnaires, may therefore underestimate prevalence and severity of sleep disturbance thereby precluding appropriate treatment.^7^

Objective remote monitoring solutions, including wearables (e.g., actigraphy, EEG headbands) and more passive (e.g., mattress) sensors have shown promise for use at home by patients with MCI and early dementia.^30^, ^31^, ^52^, ^53^ However, where resources are limited, a subjective measure more closely reflecting core objective sleep parameters for people living with cognitive impairment suitable for use in memory clinics is needed.^54^ For example, the following questions could allow for TST and WASO to be directly asked about and a potentially more accurate SE and SL to be calculated: (a) How long did it take you to fall asleep once you started trying to sleep? (b) What time did you fall asleep? (c) How long were you awake during the night (during the period you were trying to sleep)? and (d) What time did you wake up (and stop trying to get back to sleep)? These questions may be less burdensome than traditional subjective sleep measures and should be explored in future research.

While our study was not designed to determine the mechanism underlying subjective‐objective sleep discrepancy, cognitive impairment could contribute in various ways, including impaired recall, dyscalculia affecting sleep‐wake calculations, anosognosia, inaccurate time perception, and impaired executive function.^55^, ^56^ As positive night‐by‐night (episodic) sleep discrepancy was not identified in patient groups, impairments in delayed recall over a longer period or dyscalculia may be more likely than anosognosia to underlie the discrepancy. Our finding that objective SE predicted subjective sleep quality differences in all participants on a night‐by‐night (episodic) basis, but for habitual sleep quality only in controls, further supports this hypothesis. Higher variability in night‐by‐night (episodic) sleep discrepancy in the patient groups also indicates their subjective sleep estimates may be more sensitive to intra‐individual factors. Intra‐individual variability in sleep discrepancy has been associated with sleep effort, cognitive arousal, and mood upon awakening.^57^ The mechanisms underlying sleep discrepancy may also differ by underlying etiology. Observing sleep discrepancy in both AD and LBD, but worse subjective sleep quality in LBD, may be related to different underlying patterns of neurodegeneration, with early AD typically characterized by memory deficits and medial temporal cortex neurodegeneration and LBD characterized by early neurodegeneration in subcortical structures associated with movement, attention, and perception. Probing these putative mechanisms and determining which parameters predict episodic and habitual sleep discrepancy in older adults with cognitive impairment would be a valuable focus of future research.

Sleep discrepancy has been discussed as “misperception” or “error,”^23^, ^58^ which could discourage collection of subjective measures and imply low value. However, subjective sleep measures provide a reflection of overall sentiment toward sleep quality that may be important; subjective sleep measures could identify those who may be most motivated to engage in sleep interventions, and subjective sleep quality has also been found to be more closely linked to quality of life than objective sleep.^59^ Moreover, sleep discrepancy may have diagnostic utility not only for AD and LBD but also in patients with subjective or functional cognitive impairment. Individuals with subjective cognitive symptoms attending a cognitive disorders clinic reporting sleep disturbance were less likely to experience future cognitive deterioration,^60^ suggesting at least some may have a functional presentation. In further work, while self‐reported sleep problems were associated with self‐reported cognitive decline, depressive symptoms, and anxiety, there was no association with objective cognition or neurodegenerative markers.^61^

Objective sleep measures are not necessarily free from bias, particularly in patients with more sleep disturbance.^62^ Immobility during quiet wakefulness or attempting to sleep could be misinterpreted as sleep and result in shorter SL and higher SE, or loss of muscle atonia in patients with RBD may cause REM sleep to be misclassified as WASO. Differences in sleep parameters recorded by different objective measures highlight the issue in interpreting “objective” as “accurate.”^63^, ^64^, ^65^ Though generally trends were in the same direction, we observed differences in effect size between parameters measured by EEG and actigraphy.

To calculate reliable averages of core sleep parameters using objective measures, we propose that actigraphy is useful. EEG enables deeper sleep profiling than actigraphy, but it is burdensome, requiring additional set‐up time and charging. Actigraphy enables passive, user‐friendly, longer‐duration continuous data collection, potentially compensating for increased noise.^66^ However, sleep monitoring is rapidly evolving. Greater attention to how we operationalize and measure sleep, continued work to improve passive sensors and wearables, and integrating multimodal objective and subjective sleep and other biosensor health data such as through artificial intelligence models could transform how accurately and comprehensively we will be able to assess sleep in the future.^67^, ^68^

### 4.1 Limitations and future directions

Several limitations are acknowledged. Wrist actigraphy relies on movement and algorithmic detection of sleep and is typically supported by sleep diaries or event markers to improve accuracy, particularly for SL and SE.^69^ We used sleep diaries to guide the GGIR algorithm for actigraphy analysis of habitual sleep discrepancy in this study. Although sensitivity analyses relying solely on algorithmic detection identified similar results, actigraphy may not be considered a pure objective measure.^31^ Some participants also reported receiving support from relatives, including answering sleep diary questions,^31^ which may have increased the accuracy of sleep diaries compared to patients who completed them independently, potentially affecting episodic sleep discrepancy.^31^ Further, to assess habitual sleep discrepancy, participants retrospectively self‐reported sleep for 1 month prior to baseline, while actigraphy occurred after baseline. Sleep may have differed during these non‐overlapping periods. However, the PSQI is designed to capture current sleep patterns, and actigraphy data were averaged across the following 8 weeks, so direct comparison was considered reasonable.

Our analysis included patients at relatively mild stages of cognitive impairment due to two of the most common neurodegenerative conditions, AD and LBD. While participants were grouped by clinical diagnosis, limited sample size prevented analysis on whether stage of cognitive impairment or domain‐specific cognitive impairment affected sleep discrepancy within cohorts.^18^ Future work should identify whether different sleep discrepancy profiles exist across the cognitive continuum, whether sleep discrepancy could predict future cognitive decline in individuals with sleep disturbances,^25^ and whether sleep discrepancy differs by different disease etiologies, including vascular and mixed dementias.

While participants had low self‐reported neuropsychiatric symptoms, individuals misclassified by the model had higher self‐reported depression and anxiety and trended toward worse subjective sleep. Subclinical late‐life anxiety and depression have been associated with worse sleep quality and daytime sleepiness.^70^ Therefore, future studies should examine whether sleep discrepancy can differentiate between cohorts when mood disturbance is higher and consider whether mood disturbance may improve predictive models. Finally, the study power was limited. Sample size was also insufficient to allow for a training and predictive dataset; our results pertaining to diagnostic potential therefore require cautious interpretation but nonetheless warrant further investigation. Participants were also predominantly male and White British. Replicating study findings in a larger, more balanced cohort in terms of sex and ethnicity would be desirable.

## 5 CONCLUSION

Sleep disturbance likely has a bidirectional relationship with neurodegenerative diseases and dementia.^71^ However, sleep is not routinely assessed in memory clinics. Our findings contribute to the growing body of literature highlighting the potential benefits of routine sleep screening in memory clinics using questionnaires and wearable technology that can be administered at scale.^72^, ^73^, ^74^ Subjective sleep appraisal and objective sleep parameters differ in older adults with MCI or dementia due to AD or LBD but, when combined, more comprehensively profile sleep with potential diagnostic utility. Therefore, we recommend that sleep assessment in memory clinic populations and dementia research prioritize collecting subjective and objective measures and not use one as a proxy for the other.

## AUTHOR CONTRIBUTIONS

**Victoria Grace Gabb**: Conceptualization; methodology; investigation; formal analysis; resources; data curation; writing—original draft; writing—review and editing; project administration. **Jonathan Blackman**: Conceptualization; methodology; investigation; software; formal analysis; resources; writing—original draft; writing—review and editing; visualization; funding acquisition; project administration. **Hamish Duncan Morrison**: Conceptualization; methodology; investigation; resources; writing—review and editing; funding acquisition; project administration. **Nicholas Turner**: Supervision; writing—review and editing; funding acquisition. **Elizabeth Coulthard**: Conceptualization; methodology; resources; writing—review and editing; supervision; funding acquisition.

## CONFLICT OF INTEREST STATEMENT

The funders had no role in the study design, execution of the study, interpretation of findings, or writing of this paper. E.C. has received funding from Biogen, Eisai, and Lilly for consultancy and providing education resources. A.H. has received funding from Quanterix Corp. for consultancy. All other authors declare no conflicts of interest. Author disclosures are available in the Supporting Information.

## CONSENT STATEMENT

The study received approval from the Yorkshire & The Humber—Bradford Leeds Research Ethics Committee on September 13, 2021 (21/YH/0177) and was carried out in accordance with the Declaration of Helsinki. All participants provided written informed consent for participation prior to completing any study activities.

## Supporting information



Supporting Information

Supporting Information
