# Supplementary material for: Mind the gap: obtaining reliable sleep estimates and the diagnostic value of sleep discrepancy in individuals with Alzheimer's disease and Lewy body disease
Source: Alzheimers Dement. 2026 Apr 23;22(4):e71377. doi: 10.1002/alz.71377 (PMC13106027; doi:10.1002/alz.71377)
Supplement: Supplementary file 1 — Supporting Information [file ALZ-22-e71377-s002.docx]

Mind the Gap: Obtaining Reliable Sleep Estimates and The Diagnostic Value of Sleep Discrepancy in Individuals with Alzheimer's Disease and Lewy Body Disease – **Supplementary Materials**

## Appendix 1 – Full eligibility criteria for the RESTED study.

| Inclusion Criteria | |
| --- | --- |
| 1 | Age > 50 |
| 2 | All participants must express that they are willing to take part in the study and adhere to the study procedures |
| 3 | Full capacity to consent to involvement |
| 4 | Clinical diagnosis of MCI/mild dementia due to AD according to standardised criteria (AD cohort) OR  Clinical diagnosis of MCI/mild dementia due to LBD according to standardised criteria (LBD cohort) OR  Age-matched cognitively healthy adults (Control cohort) |
| Exclusion Criteria | |
| 1 | Severe medical or psychiatric co-morbidity, which, in the opinion of the investigator, may substantially impact on sleep |
| 2 | Clinically significant, severe sleep disorder as defined by ICD-10 or equivalent pre-dating and / or not related to AD or LBD pathology. |
| 3 | Diagnosis of dementia other than AD or LBD |
| 4 | Montreal Cognitive Assessment (MoCA) Total Score < 11/30 |

**Abbreviations:** RESTED – Remote Evaluation of Sleep To enhance understanding of Early Dementia; AD – Alzheimer’s disease; LBD – Lewy body disease.

## Appendix 2 – Actigraphy Algorithm Configuration for *GGIR*

Key parameters specified for GGIR algorithm:

GGIR(mode=c(1,2,3,4,5),

windowsizes = c(5,1800,5400),

nonwear_approach = "2013",

overwrite=TRUE,

HASPT.algo="vanHees2015",

HASPT.ignore.invalid=NA,

ignorenonwear = FALSE,

relyonguider = FALSE,

nonWearEdgeCorrection=TRUE,

visualreport_without_invalid = FALSE,

sleepwindowType = "TimeInBed")

## Appendix 3 – Further Information on Data Collection and Data Quality

### Dreem 2 EEG Headband

257 nights of data across 40 participants were recorded using the Dreem 2 EEG headband. A threshold of 70-85% calculated record quality has been described as sufficient for automated analysis of macro-architectural variables comparable to polysomnography. Recordings with <70% record quality were therefore discarded leaving a total of 211 total nights. A further two nights were excluded from separate participants where outlying, implausible results were returned e.g. 0 minutes WASO with TST > 10 hours. In total n=209 nights were entered for analysis.

Mean [SD] nights per participant; Full cohort, 5.5 [1.8]; AD, 4.70 [2.2]; LBD, 6.00 [0.9], Control, 5.68 [1.9]; AD vs Control p=0.240, LBD vs Control p=0.545, AD vs LBD p=0.105). One control participant did not produce a recording with > 40% quality and was therefore excluded from EEG analysis leaving a total of n=29 participants with analysable data. Mean [SD] recording quality percentage of records entered for analysis was as follows:- Full cohort; 92.6 [8.3]; AD, 91.1 [8.6]; LBD, 90.6 [8.8]; Control, 94.3 [7.7]; AD vs Control p=0.030), LBD vs Control p=0.009, AD vs LBD p=0.774.

### Actigraphy (Axivity AX3)

A total of 2332 nights of data across the full cohort of 40 participants was collected. 189 of these nights contained no data either at the beginning or the end of the downloaded file and were therefore removed. Following visual inspection, a further 9 nights were removed due to sustained non-wear.

37 diary entries were imputed due to poor HDCZA algorithm detection of the sleep period and one diary entry corrected. Finally, the sleep analysis software was instructed to solely rely on diary information in 166 nights due to clear misclassification of sleep period.

Total nights for analysis numbered 2134. Mean [SD] nights per participant; Full cohort, 53.3 [8.0]; AD, 51.5 [14.0]; LBD, 52.2 [6.8]; Control, 54.9 [3.4]; AD vs Control p=0.473, LBD vs Control p=0.270, AD vs LBD p=0.889).

### Indicative Minimum Detectible Effect (TST in AD vs. LBD)

Statistics.Tools was used for a power calculation. Based on an anticipated TST standard deviation of 54 minutes (Saint-Maurice et al., 2024), 80% power and alpha 0.05, in a two-sample comparison of n=10 individuals, the minimum detectable effect is 67.7 minutes between cohort.

P.F. Saint-Maurice, J.R. Freeman, D. Russ, J.S. Almeida, M.M. Shams-White, S. Patel, et al. Associations between actigraphy-measured sleep duration, continuity, and timing with mortality in the UK Biobank. Sleep 2024;47(3):zsad312. doi: 10.1093/sleep/zsad312

## Appendix 4 – Supplementary Results

### Subjective Sleep Quality by Cohort

Ordinal Regression Model of PSQI Subjective Sleep sub-component score and associations with cohort (AD, LBD, or control) membership

| Parameter | Estimate | Odds Ratio  [95% CI] | Std. Error | z value | p |
| --- | --- | --- | --- | --- | --- |
| 0\|1 | -3.90664 | - | 1.207494 | -3.23533 | 0.001215 |
| 1\|2 | -0.15141 | - | 1.004676 | -0.15071 | 0.880204 |
| 2\|3 | 2.173416 | - | 1.199747 | 1.811561 | 0.070054 |
| AD Cohort | -3.04924 | 0.047 (0.005, 0.43) | 1.124716 | -2.71112 | 0.006706 |
| LBD Cohort | 1.476094 | 4.376 (0.734, 26.1) | 0.91064 | 1.620941 | 0.10503 |
| GAD-7 Total | 1.294701 | 1.368 (1.08, 1.74) | 0.501838 | 2.579921 | 0.009882 |
| GDS Total | 0.475776 | 1.187 (0.784, 1.8) | 0.58785 | 0.80935 | 0.418314 |
| Age | -0.23625 | 0.961 (0.827, 1.12) | 0.455273 | -0.51892 | 0.603815 |
| Alarm clock use | -1.79865 | 0.166 (0.025, 1.08) | 0.957925 | -1.87765 | 0.060429 |
| OSA Marker | -0.95551 | 0.385 (0.066, 2.25) | 0.900608 | -1.06096 | 0.288709 |
| Sedative Use | -1.21711 | 0.296 (0.017, 5.22) | 1.463731 | -0.83151 | 0.405685 |
| Male Sex | -1.43571 | 0.238 (0.039, 1.44) | 0.917633 | -1.56458 | 0.117682 |

**Abbreviations:** AD – Alzheimer’s disease, LBD – Lewy body disease, Std. – Standard, p – p-value, OSA – Obstructive Sleep Apnoea, GAD – Generalised Anxiety Disorder, GDS – Geriatric Depression Scale, Std – Standard, p – p-value

Alarm clock use was recorded in a bespoke questionnaire on sleep at baseline.

### Predictors of Poor Self-Reported Sleep Quality

#### PSQI Subjective Sleep Quality vs Actigraphy Data

The following table depicts ordinal regression crude and adjusted models utilising the subjective sub-component of the PSQI as the dependent variable and actigraphy derived TST, SL and SE as dependent variables with interaction terms for cohort membership. Adjusted models incorporate potential confounders.

|  | **Crude Model** | | | | **Adjusted Model** | | | |
| --- | --- | --- | --- | --- | --- | --- | --- | --- |
| **Parameter** | Est. | Odds Ratio (95% CI) | SE | p | Est. | Odds Ratio (95% CI) | SE | p |
| Total Sleep Time | 1.726 | 5.618 (0.828, 38.1) | 0.98 | 0.077 | 1.414 | 4.11 (0.445, 38) | 1.13 | 0.213 |
| AD * Total Sleep Time | -2.577 | 0.07598 (0.0034, 1.7) | 1.59 | 0.104 | -1.943 | 0.1433 (0.00208, 9.9) | 2.16 | 0.369 |
| LBD * Total Sleep Time | -2.290 | 0.1012 (0.00611, 1.68) | 1.43 | 0.110 | -1.554 | 0.2114 (0.00543, 8.22) | 1.87 | 0.405 |
| Sleep Efficiency | -2.486 | 0.08328 (0.00893, 0.777) | 1.14 | 0.029* | -1.674 | 0.1876 (0.0113, 3.11) | 1.43 | 0.243 |
| AD * Sleep Efficiency | 2.749 | 15.63 (0.772, 316) | 1.53 | 0.073 | 3.021 | 20.51 (0.524, 803) | 1.87 | 0.106 |
| LBD * Sleep Efficiency | 3.009 | 20.27 (0.954, 431) | 1.56 | 0.054 | 1.358 | 3.888 (0.0629, 240) | 2.10 | 0.519 |
| Log(Sleep Latency) | -0.028 | 0.9719 (0.39, 2.42) | 0.47 | 0.951 | -0.733 | 0.4805 (0.146, 1.58) | 0.61 | 0.228 |
| AD * Log(Sleep Latency) | -0.532 | 0.5874 (0.0825, 4.18) | 1.00 | 0.595 | 1.401 | 4.059 (0.226, 72.7) | 1.47 | 0.341 |
| LBD * Log(Sleep Latency) | -0.865 | 0.4211 (0.0171, 10.3) | 1.63 | 0.596 | -1.676 | 0.1871 (0.00223, 15.7) | 2.26 | 0.458 |
| AD Cohort Membership | -1.805 | 0.1644 (0.0171, 1.58) | 1.15 | 0.118 | -3.485 | 0.03066 (0.00142, 0.662) | 1.57 | 0.026* |
| LBD Cohort Membership | 1.500 | 4.484 (0.721, 27.9) | 0.93 | 0.108 | 2.335 | 10.33 (1.11, 96) | 1.14 | 0.040* |
| Age | - |  | - | - | 0.148 | 1.16 (0.326, 4.13) | 0.65 | 0.819 |
| GAD-7 Total | - |  | - | - | 1.599 | 4.946 (1.46, 16.8) | 0.62 | 0.010* |
| GDS Total | - |  | - | - | 0.453 | 1.573 (0.399, 6.2) | 0.70 | 0.517 |
| Alarm Clock Use | - |  | - | - | -1.611 | 0.1998 (0.0195, 2.04) | 1.19 | 0.175 |
| Male Sex | - |  | - | - | -1.925 | 0.1459 (0.0155, 1.37) | 1.14 | 0.092 |
| Possible Untreated OSA | - |  | - | - | -1.280 | 0.278 (0.0309, 2.5) | 1.12 | 0.253 |
| Sedative Medication Use | - |  | - | - | -1.639 | 0.1943 (0.00257, 14.7) | 2.21 | 0.458 |

Ordinal regression of subjective subcomponent of PSQI scale in crude and adjusted models. Higher values of dependent variable representative of poorer quality sleep. Scaled / normalised values used for all continuous independent variables. Alarm clock use was recorded in a bespoke questionnaire on sleep at baseline.

**Abbreviations:** AD – Alzheimer’s disease, LBD – Lewy body disease, Std. – Standard, p – p-value, OSA – Obstructive Sleep Apnoea, GAD – Generalised Anxiety Disorder, GDS – Geriatric Depression Scale. Est – Estimate, SE – Standard Error

#### Consensus Sleep Diary Nightly Subjective Rating vs EEG data

|  | **Crude Model** | | | | **Adjusted Model** | | | |
| --- | --- | --- | --- | --- | --- | --- | --- | --- |
| **Parameter** | Est. | Odds Ratio (95% CI) | SE | p | Est. | Odds Ratio (95% CI) | SE | p |
| Total Sleep Time | 0.299 | 1.349 (0.741, 2.46) | 0.31 | 0.328 | 0.385 | 1.469 (0.805, 2.68) | 0.31 | 0.211 |
| AD * Total Sleep Time | 0.519 | 1.681 (0.6, 4.71) | 0.53 | 0.323 | 0.385 | 1.47 (0.535, 4.04) | 0.52 | 0.455 |
| LBD * Total Sleep Time | -1.012 | 0.3633 (0.159, 0.831) | 0.42 | 0.016 | -1.116 | 0.3277 (0.144, 0.743) | 0.42 | 0.008 |
| Sleep Efficiency | 1.308 | 3.698 (1.98, 6.89) | 0.32 | <0.001 | 1.32 | 3.744 (2.00, 6.99) | 0.32 | <0.001 |
| AD * Sleep Efficiency | 0.308 | 1.361 (0.421, 4.4) | 0.6 | 0.607 | 0.585 | 1.795 (0.572, 5.64) | 0.58 | 0.316 |
| LBD * Sleep Efficiency | -0.245 | 0.7824 (0.292, 2.1) | 0.5 | 0.626 | -0.268 | 0.7646 (0.293, 2) | 0.49 | 0.584 |
| Log(Sleep Latency) | 0.332 | 1.394 (0.817, 2.38) | 0.27 | 0.223 | 0.473 | 1.605 (0.957, 2.69) | 0.26 | 0.073 |
| AD * Log(Sleep Latency) | 0.343 | 1.409 (0.414, 4.8) | 0.63 | 0.584 | 0.371 | 1.449 (0.465, 4.51) | 0.58 | 0.522 |
| LBD * Log(Sleep Latency) | -0.481 | 0.6184 (0.224, 1.71) | 0.52 | 0.353 | -0.511 | 0.5999 (0.223, 1.61) | 0.5 | 0.311 |
| AD Cohort Membership | 1.347 | 3.847 (0.817, 18.1) | 0.79 | 0.088 | 2.287 | 9.845 (2.25, 43.1) | 0.75 | 0.002 |
| LBD Cohort Membership | 1.15 | 3.158 (0.722, 13.8) | 0.75 | 0.126 | 0.553 | 1.739 (0.466, 6.49) | 0.67 | 0.41 |
| Age | - |  | - | - | 1.245 | 3.474 (1.74, 6.93) | 0.35 | <0.001 |
| GAD-7 Total | - |  | - | - | -0.544 | 0.5803 (0.296, 1.14) | 0.34 | 0.113 |
| GDS Total | - |  | - | - | -0.475 | 0.622 (0.265, 1.46) | 0.44 | 0.276 |
| Alarm Clock Use | - |  | - | - | -0.057 | 0.9442 (0.273, 3.27) | 0.63 | 0.928 |
| Male Sex | - |  | - | - | 0.496 | 1.642 (0.45, 5.99) | 0.66 | 0.453 |
| Possible Untreated OSA | - |  | - | - | 0.831 | 2.295 (0.623, 8.45) | 0.67 | 0.212 |
| Sedative Medication Use | - |  | - | - | 0.392 | 1.48 (0.161, 13.6) | 1.13 | 0.729 |

Ordinal regression of subjective subcomponent of PSQI scale in crude and adjusted models. Higher values of dependent variable representative of poorer quality sleep. Scaled / normalised values used for all continuous independent variables.

**Abbreviations:** AD – Alzheimer’s disease, LBD – Lewy body disease, Std. – Standard, p – p-value, OSA – Obstructive Sleep Apnoea, GAD – Generalised Anxiety Disorder, GDS – Geriatric Depression Scale, EEG – electroencephalography using Dreem 2

### Nightly Consensus Sleep Diary versus EEG-derived Sleep Parameters

| **Coefficient** | **Estimate** | **Std. Error** | **Lower 95% CI** | **Upper 95% CI** |
| --- | --- | --- | --- | --- |
| Total Sleep Time |  |  |  |  |
| Intraclass Correlation | 43.42* | 7.93 | 29.05 | 61.02 |
| Intercept | -21.04* | 10.73 | -42.49 | -0.54 |
| AD | 4.47 | 20.6 | -36.4 | 45.13 |
| LBD | 18.65 | 22.39 | -25.36 | 63.14 |
| Sigma Intercept | 3.6* | 0.08 | 3.44 | 3.76 |
| Sigma AD | 0.55* | 0.14 | 0.28 | 0.83 |
| Sigma LBD | 0.97* | 0.14 | 0.71 | 1.25 |
| Sleep Latency |  |  |  |  |
| Intraclass Correlation | 10.68* | 2.54 | 6.03 | 16 |
| Intercept | 1.79 | 3.32 | -4.91 | 8.37 |
| AD | 13.08* | 5.59 | 2.57 | 24.4 |
| LBD | -4.96 | 6.47 | -17.12 | 8.43 |
| Sigma Intercept | 3.05* | 0.08 | 2.91 | 3.21 |
| Sigma AD | -0.28* | 0.14 | -0.54 | -0.003 |
| Sigma LBD | 0.28* | 0.13 | 0.02 | 0.56 |
| Sleep Efficiency |  |  |  |  |
| Intraclass Correlation | 9.57* | 1.37 | 7.27 | 12.63 |
| Intercept | 2.08 | 2.32 | -2.46 | 6.75 |
| AD | 0.57 | 4.3 | -7.87 | 9.13 |
| LBD | -1.74 | 4.19 | -9.84 | 6.72 |
| Sigma Intercept | 1.94* | 0.08 | 1.79 | 2.1 |
| Sigma AD | 0.32* | 0.14 | 0.05 | 0.6 |
| Sigma LBD | 0.35* | 0.13 | 0.09 | 0.61 |

**Abbreviations:** AD – Alzheimer’s disease, LBD – Lewy body disease,SE – Standard Error, CI – Confidence Interval, EEG – electroencephalography using Dreem 2

### Stable Mean Reliability of Actigraphy vs EEG-derived Sleep Parameters

The number of measurements required to achieve acceptable, good and excellent sleep parameter mean reliability by cohort for EEG and actigraphy is shown in the table below.

|  |  | **Full** |  |  | **AD** |  |  | **LBD** |  | **Control** | | |
| --- | --- | --- | --- | --- | --- | --- | --- | --- | --- | --- | --- | --- |
|  | R value | | | R value | | | R value | | | R value | | |
|  | 0.7 | 0.8 | 0.9 | 0.7 | 0.8 | 0.9 | 0.7 | 0.8 | 0.9 | 0.7 | 0.8 | 0.9 |
| **EEG** |  |  |  |  |  |  |  |  |  |  |  |  |
| TST | 10.6 | 18.2 | 41 | 6 | 10.3 | 23.1 | 15.6 | 26.8 | 60.2 | 15.6 | 26.8 | 60.2 |
| SL | 2.4 | 4.2 | 9.4 | 2.9 | 4.9 | 11 | 4.3 | 7.4 | 16.7 | 1.6 | 2.8 | 6.3 |
| SE | 3.8 | 6.5 | 14.7 | 4.1 | 7.1 | 16 | 4.5 | 7.8 | 17.5 | 3.6 | 6.3 | 14.1 |
| WASO | 4.7 | 8.1 | 18.3 | 3 | 5.1 | 11.5 | 5 | 8.5 | 19.1 | 7.8 | 13.4 | 30.1 |
| **Act.** |  |  |  |  |  |  |  |  |  |  |  |  |
| TST | 3.4 | 5.8 | 13 | 5.2 | 8.9 | 20 | 2.9 | 4.9 | 11 | 3.4 | 5.8 | 13 |
| SL | 32 | 54.8 | 123.4 | 47.3 | 81.1 | 182.5 | 143.5 | 246 | 553.5 | 23.3 | 40 | 89.9 |
| SE | 2.5 | 4.3 | 9.8 | 3 | 5.1 | 11.5 | 1.6 | 2.7 | 6 | 4.3 | 7.4 | 16.7 |
| WASO | 2.6 | 4.5 | 10.1 | 6.3 | 10.8 | 24.3 | 1.4 | 2.3 | 5.3 | 4 | 6.8 | 15.3 |

**Abbreviations:** AD – Alzheimer’s disease, LBD – Lewy body disease, EEG – electroencephalography using Dreem 2, Act. – actigraphy using Axivity AX3, TST – Total Sleep Time, SL – Sleep Latency, SE – Sleep Efficiency, WASO – Wake After Sleep Onset.

### Mean Subjective / Objective Discrepancy by Cohort – EEG Data

Subjective (PSQI) vs Objective (EEG-derived) Sleep Parameters by Cohort

| Discrepancy (mins) | AD Mean (SD) | LBD Mean (SD) | Cont. Mean (SD) | AD vs Cont Cohen's d (95% CI) | AD vs Cont p* | LBD vs Cont Cohen's d (95% CI) | LBD vs Cont p* | AD vs LBD Cohen's d (95% CI) | AD vs LBD p* |
| --- | --- | --- | --- | --- | --- | --- | --- | --- | --- |
| TST | 53.7  (50) | 17.9  (118) | 1.24  (73) | 0.791  (-0.04, 1.6) | 0.032 | 0.187  (-0.65, 1) | 0.704 | 0.404  (-0.58, 1.4) | 0.417 |
| SL | -18.3  (14) | -0.312  (42) | 1.82  (25) | -0.904  (-1.7, -0.07) | 0.011 | -0.0675  (-0.9, 0.76) | 0.892 | -0.584  (-1.6, 0.41) | 0.253 |
| SE | -1.2  (8.9) | 0.088  (19) | -7.64  (15) | 0.491  (-0.32, 1.3) | 0.156 | 0.481  (-0.36, 1.3) | 0.296 | -0.090  (-1.1, 0.9) | 0.854 |

Discrepancy metrics calculated by subtracting EEG-derived mean sleep parameters from subjective, self-report PSQI equivalent.

**Abbreviations:** AD – Alzheimer’s disease, LBD – Lewy body disease, Cont. – Control, SE – Standard Error, CI – Confidence Interval, EEG – electroencephalography using Dreem 2, Act. – actigraphy using Axivity AX3

### Crude and Adjusted Linear Mixed Effects Models demonstrating objective (actigraphy-derived) vs subjective (PSQI-derived) sleep discrepancy by cohort and sleep parameter

|  | **Crude Model** | | | **Adjusted Model** | | |
| --- | --- | --- | --- | --- | --- | --- |
| Parameter | Estimate | Std. Error | p value | Estimate | Std. Error | p value |
| Actigraphy TST Discrepancy (mins) | | | | | | |
| AD | 88.1 | 28 | 0.003 | 120 | 30 | <0.001 |
| LBD | 77.5 | 28 | 0.009 | 62.1 | 29 | 0.04 |
| Actigraphy SL Discrepancy (log mins) | | | | | | |
| AD | -0.988 | 0.517 | 0.064 | -1.41 | 0.603 | 0.026 |
| LBD | 0.136 | 0.517 | 0.794 | -0.124 | 0.584 | 0.834 |
| Actigraphy SE Discrepancy (%) | | | | | | |
| AD | 10.2 | 6.87 | 0.147 | 15 | 7.85 | 0.066 |
| LBD | 12.7 | 6.87 | 0.072 | 10.1 | 7.6 | 0.194 |
| Actigraphy WASO Discrepancy (mins) | | | | | | |
| AD | -26.2 | 36.5 | 0.478 | -38.3 | 42.5 | 0.374 |
| LBD | -76.2 | 36.5 | 0.044 | -58.6 | 41.2 | 0.165 |
| PSQI Subjective Sleep Component | | | | | | |
| AD | -0.5 | 0.291 | 0.094 | -0.758 | 0.299 | 0.016 |
| LBD | 0.4 | 0.291 | 0.178 | 0.395 | 0.289 | 0.182 |

**Abbreviations:** AD – Alzheimer’s disease, LBD – Lewy body disease, GDS – Geriatric Depression Scale, GAD – Generalised Anxiety Disorder, PSQI – Pittsburgh Sleep Quality Index, Std. Error – Standard Error.

Estimates produced by linear regression with AD and LBD cohort memberships modelled by dummy variables indicative of AD and LBD cohort membership respectively. Models produced for total sleep time (TST) discrepancy = (PSQI sleep duration – Mean Actigraphy TST), sleep latency (SL) discrepancy = (log (PSQI sleep latency) – log (mean actigraphy latency)), sleep efficiency (SE) discrepancy = (PSQI sleep efficiency – mean actigraphy SE). Adjusted models control for age, gender, GDS (Geriatric Depression Scale), GAD-7 (Generalised Anxiety Disorder 7), presence of possible untreated OSA and use of sedative / sleep medications.

Note: Sleep latency dependent variable log transformed to maintain assumptions for linear regression modelling correcting for high residual kurtosis.

### Linear Regression Modelling of associations between subjective (PSQI-derived) vs objective (actigraphy-derived) discrepancy and plasma neurodegenerative biomarkers

|  | **Actigraphy** | | |
| --- | --- | --- | --- |
|  | Est. | Std. Error | p-value |
|  | Scaled (Aβ42 / 40 Ratio) | | |
| TST Over-Estimation, mins | -28.6 | 13.9 | 0.049^a^ |
| SL Over-Estimation, log difference | 0.0108 | 0.253 | 0.966 |
| SE Over-Estimation, % | -3.67 | 3.27 | 0.272 |
|  | Scaled (p-tau 217) | | |
| TST Over-Estimation, mins | 35.4 | 15 | 0.026^b^ |
| SL Over-Estimation, log difference | -0.275 | 0.274 | 0.324 |
| SE Over-Estimation, % | 6.93 | 3.43 | 0.054 |
|  | Scaled (p-tau 181 / Aβ42 Ratio) | | |
| TST Over-Estimation, mins | 14.7 | 16.3 | 0.377 |
| SL Over-Estimation, log difference | 0.0604 | 0.278 | 0.830 |
| SE Over-Estimation, % | 4.14 | 3.62 | 0.263 |
|  | Scaled Neurofilament Light (NfL) | | |
| TST Over-Estimation, mins | 32.5 | 15.3 | 0.044^c^ |
| SL Over-Estimation, log difference | -0.566 | 0.258 | 0.037^d^ |
| SE Over-Estimation, % | 2.01 | 3.7 | 0.592 |
|  | Scaled GFAP | | |
| TST Over-Estimation, mins | 17 | 16 | 0.297 |
| SL Over-Estimation, log difference | -0.499 | 0.258 | 0.065 |
| SE Over-Estimation, % | 3.71 | 3.59 | 0.311 |

**Abbreviations:** Std. Error – Standard Error, TST – total sleep time, SL – sleep (onset) latency, SE – sleep efficiency, Aβ – amyloid-beta, p-tau – phosphorylated tau, GFAP – Glial Fibrillary Acidic Protein

Separate linear regression models with TST, SL, and SE as dependent variables and Aβ 42 / 40 Ratio, p-tau 217, p-tau 181 / Aβ42 Ratio, NFL and GFAP as independent (predictor) variables controlling for age, gender, GDS (Geriatric Depression Scale), GAD-7 (Generalised Anxiety Disorder-7), presence of possible untreated OSA and use of sedative medications:

^a^ p=0.049 > p=0.030 (Corrected p value with FDR n=5).

^b^ p=0.026 > p=0.010 (Corrected p value with FDR n=5).

^c^ p=0.044 > p=0.020 (Corrected p value with FDR n=5).

^d^ p=0.037 > p=0.010 (Corrected p value with FDR n=5).

### Correctly vs Incorrectly Identified Individuals Stratified by Cohort

#### Comparison between individuals with AD correctly and incorrectly classified by the classification model

|  | Incorrect Classification  N=3  Mean (SD) | Correct Classification  N=7  Mean (SD) | Mean Difference (95% CI) | Cohen's d  (95% CI) | p-value |
| --- | --- | --- | --- | --- | --- |
| GDS Total | 5.67 (6.4) | 4 (2) | 1.67  (-13, 17) | 0.456  (-1.2, 2.1) | 0.700 |
| GAD-7 Total | 9.33 (7.2) | 4 (3.6) | 5.33  (-11, 21) | 1.12  (-0.57, 2.8) | 0.329 |
| Age (yrs) | 66 (13) | 70.6 (5.4) | -4.57  (-34, 25) | -0.569  (-2.2, 1) | 0.611 |
| Male Sex | 100% | 71.4% | 28.6  (-17, 74) | 0.676  (-0.95, 2.3) | 0.172 |
| PSQI Total | 4.33 (3.1) | 3.71 (1.5) | 0.62  (-6.2, 7.4) | 0.309  (-1.3, 1.9) | 0.765 |
| OSA Marker | 0% (0) | 14.3% (3.8) | -14.3  (-49, 21) | -0.436  (-2, 1.2) | 0.356 |
| Actigraphy TST (mins) | 355 (49) | 323 (56) | 31.6  (-62, 126) | 0.587  (-1, 2.2) | 0.413 |
| Actigraphy SE (%) | 78.5 (7.8) | 73.5 (12) | 4.96  (-10, 20) | 0.461  (-1.1, 2.1) | 0.459 |
| Actigraphy SL (mins) | 20.5 (5.5) | 25.7 (7.9) | -5.23  (-16, 5.7) | -0.712  (-2.3, 0.92) | 0.279 |
| Actigraphy WASO (mins) | 60.3 (41) | 67.9 (36) | -7.61  (-88, 73) | -0.205  (-1.8, 1.4) | 0.795 |

**Abbreviations:** AD – Alzheimer’s disease, SD – standard deviation, GDS – Geriatric Depression Scale, GAD – Generalised Anxiety Disorder, PSQI – Pittsburgh Sleep Quality Inventory, OSA – obstructive sleep apnoea, TST – total sleep time, SE – sleep efficiency, SL – sleep (onset) latency, WASO – wake after sleep onset.

#### Comparison between individuals with LBD correctly and incorrectly classified by the classification model

|  | Incorrect Classification  N=4  Mean (SD) | Correct Classification  N=6  Mean (SD) | Mean Difference (95% CI) | Cohen's d (95% CI) | p-value |
| --- | --- | --- | --- | --- | --- |
| GDS Total | 4.5 (0.58) | 3.83 (3.1) | 0.667  (-2.5, 3.9) | 0.273  (-1.2, 1.8) | 0.623 |
| GAD-7 Total | 3 (0.82) | 3.83 (2.6) | -0.833  (-3.6, 2) | -0.388  (-1.9, 1.1) | 0.495 |
| Age (years) | 74 (3.7) | 73.8 (2.3) | 0.167  (-5.4, 5.7) | 0.0568  (-1.4, 1.5) | 0.940 |
| Male Sex | 75% | 83.3% | -8.3  (-83, 66) | -0.187  (-1.7, 1.3) | 0.791 |
| PSQI Total | 9.25 (4.6) | 7.5 (3.1) | 1.75  (-5, 8.5) | 0.471  (-1, 2) | 0.533 |
| OSA Marker | 25% | 33.3% | -8.3  (-86, 70) | -0.163  (-1.7, 1.3) | 0.806 |
| Actigraphy TST (mins) | 365 (40) | 349 (82) | 16.4  (-74, 107) | 0.238  (-1.3, 1.7) | 0.684 |
| Actigraphy SE (%) | 76.6 (2.8) | 67.1 (17) | 9.52  (-8.1, 27) | 0.711  (-0.82, 2.2) | 0.228 |
| Actigraphy SL (mins) | 21.9 (5.2) | 20.5 (4.5) | 1.46  (-6.4, 9.3) | 0.306  (-1.2, 1.8) | 0.662 |
| Actigraphy WASO (mins) | 67.5 (21) | 129 (75) | -61.5  (-140, 17) | -1.01  (-2.6, 0.56) | 0.106 |

**Abbreviations:** LBD – Lewy body disease, SD – standard deviation, GDS – Geriatric Depression Scale, GAD – Generalised Anxiety Disorder, PSQI – Pittsburgh Sleep Quality Inventory, OSA – obstructive sleep apnoea, TST – total sleep time, SE – sleep efficiency, SL – sleep (onset) latency, WASO – wake after sleep onset.

#### Comparison between cognitively unimpaired controls correctly and incorrectly classified by the classification model

|  | Incorrect Classification  N=2  Mean (SD) | Correct Classification  N=18  Mean (SD) | Mean Difference (95% CI) | Cohen's d (95% CI) | p-value |
| --- | --- | --- | --- | --- | --- |
| GDS Total | 6 (1.4) | 1.78 (1.9) | 4.22  (-2.8, 11) | 2.25  (0.52, 4) | 0.101 |
| GAD-7 Total | 12 (4.2) | 2.61 (3.2) | 9.39  (-21, 39) | 2.84  (1, 4.7) | 0.179 |
| Age (yrs) | 77 (0) | 69.5 (5.5) | 7.5  (4.8, 10) | 1.4  (-0.24, 3) | <0.001 |
| Male Sex | 50% | 77.8% | -0.278  (-5.7, 5.1) | -0.62  (-2.2, 0.96) | 0.677 |
| PSQI Total | 8.5 (9.2) | 5.44 (3.3) | 3.06  (-75, 81) | 0.782  (-0.81, 2.4) | 0.720 |
| OSA Marker | 0.5 (0.71) | 0.222 (0.43) | 0.278  (-5.1, 5.7) | 0.62  (-0.96, 2.2) | 0.677 |
| Actigraphy TST (mins) | 369 (124) | 381 (48) | -12  (-1051, 1027) | -0.216  (-1.8, 1.4) | 0.914 |
| Actigraphy SE (%) | 76.2 (6.5) | 79.6 (7.5) | -3.39  (-40, 33) | -0.455  (-2, 1.1) | 0.594 |
| Actigraphy SL (mins) | 26 (5.9) | 16.4 (6.9) | 9.63  (-22, 42) | 1.4  (-0.23, 3) | 0.223 |
| Actigraphy WASO (mins) | 56.3 (9.4) | 61.1 (38) | -4.83  (-31, 22) | -0.131  (-1.7, 1.4) | 0.678 |

**Abbreviations:** SD – standard deviation, GDS – Geriatric Depression Scale, GAD – Generalised Anxiety Disorder, PSQI – Pittsburgh Sleep Quality Inventory, OSA – obstructive sleep apnoea, TST – total sleep time, SE – sleep efficiency, SL – sleep (onset) latency, WASO – wake after sleep onset.

### Predictive Model Statistics

|  | **AD** | **LBD** | **Control** |
| --- | --- | --- | --- |
| Sensitivity | 0.700 | 0.600 | 0.900 |
| Specificity | 0.933 | 0.967 | 0.700 |
| Positive Predictive Value | 0.778 | 0.857 | 0.750 |
| Negative Predictive Value | 0.903 | 0.879 | 0.875 |
| Prevalence | 0.250 | 0.250 | 0.500 |
| Detection Rate | 0.175 | 0.150 | 0.450 |
| Detection Prevalence | 0.225 | 0.175 | 0.600 |
| Balanced Accuracy | 0.817 | 0.783 | 0.800 |

**Abbreviations:** AD – Alzheimer’s disease, LBD – Lewy body disease
